# Dysregulated expression of death, stress and mitochondrion related genes in the sciatic nerve of presymptomatic SOD1^G93A^ mouse model of Amyotrophic Lateral Sclerosis

**DOI:** 10.3389/fncel.2015.00332

**Published:** 2015-09-01

**Authors:** Chrystian J. Alves, Jessica R. Maximino, Gerson Chadi

**Affiliations:** Department of Neurology, Neuroregeneration Center, University of São Paulo School of MedicineSão Paulo, Brazil

**Keywords:** ALS, SOD1^G93A^, pre-symptomatic, sciatic nerve, microarray, flow cytometry sorting, Schwann cells, network analysis

## Abstract

Schwann cells are the main source of paracrine support to motor neurons. Oxidative stress and mitochondrial dysfunction have been correlated to motor neuron death in Amyotrophic Lateral Sclerosis (ALS). Despite the involvement of Schwann cells in early neuromuscular disruption in ALS, detailed molecular events of a dying-back triggering are unknown. Sciatic nerves of presymptomatic (60-day-old) SOD1^G93A^ mice were submitted to a high-density oligonucleotide microarray analysis. DAVID demonstrated the deregulated genes related to death, stress and mitochondrion, which allowed the identification of Cell cycle, ErbB signaling, Tryptophan metabolism and Rig-I-like receptor signaling as the most representative KEGG pathways. The protein-protein interaction networks based upon deregulated genes have identified the top hubs (TRAF2, H2AFX, E2F1, FOXO3, MSH2, NGFR, TGFBR1) and bottlenecks (TRAF2, E2F1, CDKN1B, TWIST1, FOXO3). Schwann cells were enriched from the sciatic nerve of presymptomatic mice using flow cytometry cell sorting. qPCR showed the up regulated (*Ngfr, Cdnkn1b, E2f1, Traf2 and Erbb3, H2afx, Cdkn1a, Hspa1, Prdx, Mapk10*) and down-regulated (*Foxo3, Mtor*) genes in the enriched Schwann cells. In conclusion, molecular analyses in the presymptomatic sciatic nerve demonstrated the involvement of death, oxidative stress, and mitochondrial pathways in the Schwann cell non-autonomous mechanisms in the early stages of ALS.

## Introduction

Amyotrophic Lateral Sclerosis (ALS) is a fatal neurodegenerative disease characterized by a selective death of motor neurons of the spinal cord, brainstem and cerebral cortex, leading to progressive paralysis. The patient's death is often due to a respiratory failure, usually within 3–5 years from diagnosis (Kiernan et al., 2011).

ALS pathogenesis is poorly understood and the proposed mechanisms related to neurodegeneration seem to be multifactorial and include mitochondrial dysfunction, oxidative stress, protein aggregation and axonal transport impairment (Boillée et al., 2006a; de Vos et al., 2007). Additionally, mechanisms associated with motor neuron degeneration involve non-neuronal cells (Boillée et al., 2006a,b; Yamanaka et al., 2008; Wang et al., 2011), as seen by toxicity in neuron-glial co-culture experiments and also by the activation of molecular pathways related to paracrine stress signaling (Boillée et al., 2006a,b; Ferraiuolo et al., 2007; Kiernan et al., 2011).

Evidence obtained from studies on early axon and neuromuscular junction events in ALS patients and a mouse model have indicated the presence of peripheral motor neuron dysfunction before the activation of death pathways and the onset of clinical symptoms (Fischer et al., 2004; Gould et al., 2006; Moloney et al., 2014). Indeed, the peripheral events seem to proceed toward soma in a retrograde dying-back manner (Coleman and Perry, 2002; Fischer et al., 2004; Saxena and Caroni, 2007; Rocha et al., 2013). Oxidative stress and compromised mitochondria represent one set of proposed mechanisms underlying peripheral ALS dysfunction (Barber et al., 2006; Cozzolino and Carrì, 2012; Cozzolino et al., 2013).

The Schwann cell is the major functional cell type supporting axonal integrity (Mirsky and Jessen, 1999). Recent evidence suggests that Schwann cells may contribute to ALS distal axonopathy (Fischer et al., 2004; de Winter et al., 2006; Gorlewicz et al., 2009; Keller et al., 2009; Lobsiger et al., 2009; Chen et al., 2010; Verheijen et al., 2014). For instance, the up-regulation of inducible nitric oxide synthase and semaphorin 3A in the Schwann cells close to terminal fibers of the sciatic nerve has been associated with dying-back mechanisms in presymptomatic ALS mice (de Winter et al., 2006; Keller et al., 2009; Chen et al., 2010; Malaspina et al., 2010; Venkova et al., 2014). Furthermore, accumulation of axonal ribosomes in Schwann cells bearing mutant hSOD1 in a presymptomatic phase of ALS mouse model further suggests an early involvment of this glial cell type in the pathogenesis of the disease (Verheijen et al., 2014).

Microarray analyses have been useful in identifying new molecular cues potentially involved in ALS pathogenesis both in *postmortem* human tissue and also in several clinical stages of experimental animal models of ALS (Olsen et al., 2001; Hensley et al., 2002; Yoshihara et al., 2002; Dangond et al., 2004; Perrin et al., 2005; Ferraiuolo et al., 2007, 2009; Fukada et al., 2007; Vargas et al., 2008; Kudo et al., 2010; Boutahar et al., 2011; Cooper-Knock et al., 2012; de Oliveira et al., 2013, 2014; Maximino et al., 2014). However, there is a lack of information on gene expression in peripheral motor nerves in ALS despite the importance of recently described dying-back events in this disorder. Furthermore, an evaluation of dysregulated genes in specific, enriched cell populations obtained by cell sorting might extend these molecular analyses at cellular level.

By means of a high-density oligonucleotide microarray analysis linked to specific tools capable of identifying distinct cellular components and biological processes, the aim of this work was to determine whether the expression of genes involved in the regulation of death, stress and mitochondrial function was dysregulated in the sciatic nerve of the SOD1^G93A^ mouse model during the presymptomatic stage of ALS. This work has also evaluated the modulation of selected molecules in enriched sciatic nerve-derived Schwann cells, thus detailing the role of these glial cells in the early phase of this disease.

## Materials and methods

### Animal and tissue sample

Transgenic SOD1^G93A^ mice (The Jackson Laboratory, Bar Harbor, ME, USA) were crossbred and the colony was maintained in a specific pathogen-free environment within the animal facility of the University of São Paulo Medical School (São Paulo, Brazil) as described previously (Gurney, 1994; Scorisa et al., 2010; Alves et al., 2011). Animals were kept under controlled temperature and humidity conditions with a standardized light–dark cycle (lights on at 7:00 a.m. and off at 7:00 p.m.) and free access to food pellets and tap water. Mice were genotyped by PCR amplification of tail extracted DNA which identified the presence of the human SOD1 mutated gene (mSOD1) (Gurney, 1994; Scorisa et al., 2010; Alves et al., 2011). The Transgenic SOD1^G93A^ mice express high number of mutant human SOD1 copies (Gurney, 1994; Verheijen et al., 2014). The study was conducted under protocols approved by the Ethical Animal Care and Use Committee at the University of São Paulo and in accordance with the Guide for the Care and Use of Laboratory Animals adopted by the National Institutes of Health.

Sixty-day-old presymptomatic male SOD1^G93A^ mice and their age-paired wild-type controls (~20–25 g body weight) were used in the experiments. No motor neuron death was seen in any animal at this age (Alves et al., 2011), therefore the animals were chosen for the present presymptomatic analyses. Animals were killed by decapitation and sciatic nerves were removed, frozen and stored at −80°C for further use. Four mice were used in each group for the microarray experiments. The quantitative polymerase chain reaction (qPCR) analyses of sciatic nerves were performed using samples from six different mice from each transgene and wild-type groups.

### Immunofluorescence labelings and histological sections of sciatic nerve

Four animals per genotype were used for immunofluorescence labelings, according to previous publications (Guzen et al., 2009; Batista et al., 2014). Mice were anesthetized with sodium pentobarbital and euthanized by a transcardiac perfusion with 7 ml isotonic saline at room temperature followed by 35 ml fixation fluid (4°C) over a period of 6 min. The fixative consisted of 4% paraformaldehyde (w/v) in 0.1 M phosphate buffer (pH 6.9). The sciatic nerves were removed, kept in fixative at 4°C for 90 min and rinsed for 24 h in 10% sucrose dissolved in PBS. Sciatic nerves were then frozen in dry ice-cooled (−40°C) isopentane (Sigma) and stored at a −80°C freezer until use. Longitudinal sections (5 μm thick) were obtained using a cryostat (Leica, CM3000, Germany). The sections were initially washed for 2 × 10 min in PBS and then were incubated overnight in PBS containing 0.5% Triton X-100 (Sigma) and 1% bovine albumin serum (BSA, Sigma) with a polyclonal rabbit antibody against microtubule associated protein 2 (MAP2; diluted 1:200; Sigma), growth associated protein 43 (GAP-43; diluted 1:200; Sigma), S100 (diluted 1:200; Abcam) and p75NGF neurotrophin receptor (p75; diluted 1:200; Abcam). After the incubation of the primary antibodies, sections were washed for 2 × 10 min in PBS and incubated for 1 h in the dark at 37°C with a dilution of Alexa Fluor® 488 or 594-conjugated secondary antibodies specific for rabbit (1:200, all from Invitrogen, USA). Preparations were mounted on microscope slides and counterstained with nuclear 4′,6-diamidino-2-phenylindole dihydrochloride (DAPI; Vector, USA). Digital images were obtained by means of an Olympus BX-51 microscope (Olympus, USA).

In addition, four animals per genotype were processed to histological staining using methylene blue. Briefly, sciatic nerves were fixed in 2.5% glutaraldehyde, pH 7.4 (24 h). After extensive wash, tissues were embedded in araldite and transverse semi-thin sections (0.5 μm thickness) were obtained. The sections were stained with methylene blue and photomicrographed using an Olympus BX-51 microscope (Olympus).

### RNA isolation and microarray experiments

The procedures for RNA isolation and microarray experiments with sciatic nerve were described in our previous publication (Maximino et al., 2014). Briefly, RNAs from samples (25 ng) and reference (100 ng) were reverse transcribed by the Low-input RNA Linear Amplification kit and then transcribed to Cy3-labeled (samples) or Cy5-labeled (reference) RNAs according to the manufacturer's instructions (Agilent Technologies, USA) and to our previously described protocols (de Oliveira et al., 2013).

### Microarray analysis

Raw image data were converted to numerical data using the Agilent Feature Extraction Software, version 11.0.1.1, as described in our previous study (Maximino et al., 2014; Alves et al., 2015). Raw signal intensities were normalized using the GeneSpring GX v12.6 software package (Agilent Technologies, USA). After normalization, the probes were tested for differential expression. GeneSpring GX was also used in the statistical analyses of gene expressions between genotypes (SOD1^G93A^ × wild-type), according to previous publications (Smyth, 2004; Fu et al., 2014; Yang et al., 2014; Ryan et al., 2015; Wang et al., 2015). Genes with *p* < 0.05 were considered differentially expressed. The raw data from hybridizations are available on the Gene Expression Omnibus Database, and the GEO accession number is GSE69450.

### Bioinformatic analysis

The dysregulated genes were submitted for the following analyses to provide information regarding their involvement with specific cellular/molecular pathways related to ALS:

#### Functional enrichment analysis

The Database for Annotation, Visualization and Integrated Discovery (DAVID) v6.7b functional tool (https://david.ncifcrf.gov/) (Huang Da et al., 2008) was used to identify genes related to death, stress and mitochondrial function through the Gene Ontology (GO) annotation database. The DAVID analysis focused on the category: Biological Process and Cellular Component. Stringency (EASE score set to 0.05) parameters were selected to improve confidence in the terms designated as enriched. The Biological Process and Cellular Component terms related to death, stress and mitochondrial function were further organized using the BioVenn tool (http://www.cmbi.ru.nl/cdd/biovenn/) (Hulsen et al., 2008) which identifies common and exclusively expressed genes between lists.

In order to identify over-represented pathways, the GO terms list containing genes related to death, stress and mitochondrion was submitted to the DAVID tool using the Kyoto Encyclopedia of Genes and Genomes (KEGG).

#### Protein interaction network analysis

The Cytoscape plugin GeneMANIA (Warde-Farley et al., 2010) was used to predict protein interactions from the list of differentially expressed genes in the sciatic nerve of presymptomatic SOD1^G93A^ mice related to death, stress and mitochondrion. The network was generated using only information derived from the pathway and physical interactions categories in GeneMANIA. The connectivity of the nodes contained in the network was achieved by means of the node centrality parameters “degree” and “betweenness,” using the Cytoscape plug-in CentiScaPe (Scardoni et al., 2009). Node degree is a local structure measure in networks that determines the number of edges in each node. Conversely, betweenness centrality is a global structure measure in networks that determines the number of shortest paths passing through a specific node while connecting, directly or indirectly, pairs of nodes (Scardoni et al., 2009). A scatter plot was constructed by inputting node degree and betweenness values for each node in GraphPad Prism 5. The combination of such attributes in the scatter plot allowed the visualization of hubs (nodes with high node degree) and bottlenecks (nodes with high node betweenness). Subnetworks were built using list of genes related to death, stress and mitochondrion separately; scatter plots were also constructed by inputting node degree and betweenness values for each node in GraphPad Prism 5.

### Flow cytometry sorting of isolated schwann cells and fibroblast

**Figure 1:**
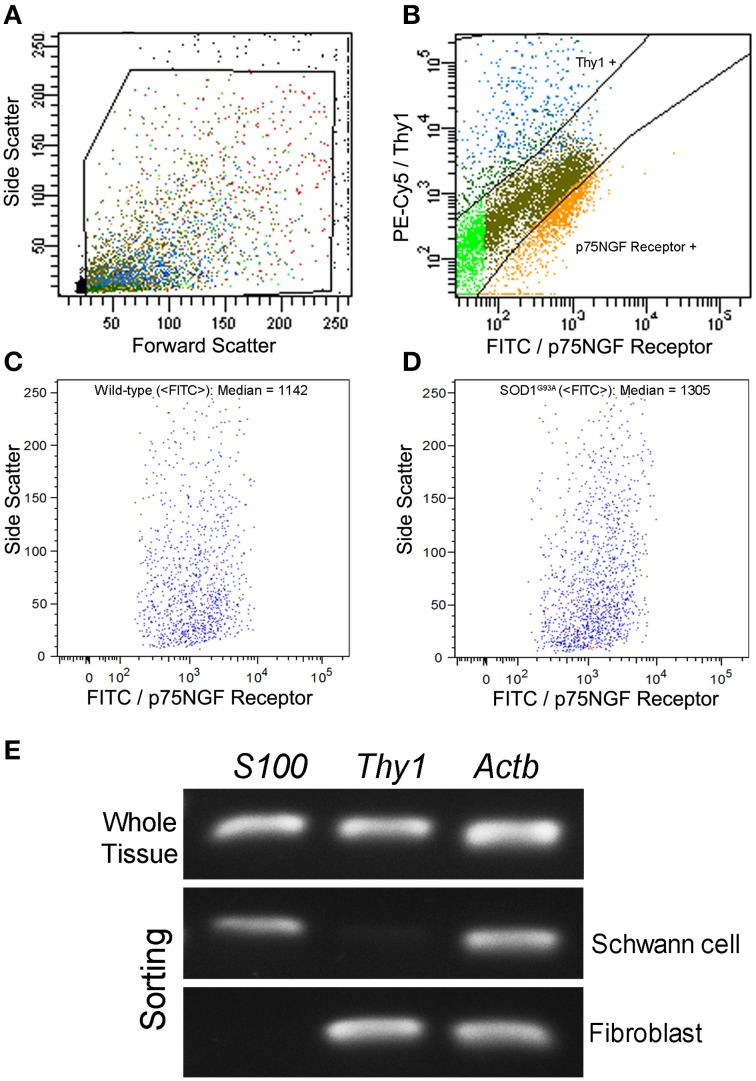
**Flow cytometry cell sorting (A–D) and PCR analysis of Schwann cell and fibroblast markers (E) from sciatic nerve of 60-day-old presymptomatic SOD1^G93A^ and the wild-type controls**. FITC-conjugated p75NGF Receptor and PE-Cy5-conjugated Thy1 antibodies were employed in the two-color immunolabeling of Schwann cells and fibroblasts, respectively **(A,B)**. Dot plots indicate the total number of events in the sciatic nerve cell suspension, and the dots inside the box represent the excluded doublet and dead profiles, which have been eliminated by morphological criteria, according to previous descriptions (Herzenberg et al., 2006) **(A)**. After morphological criteria, dot plots of Schwann cell and fibroblast profiles **(B)** were obtained using respective fluorescence filters and the blots inside the boxes represent the specific profiles after discounting the unspecific labeling. Positive p75NGF Receptor and Thy1 cell profiles are shown in corresponding boxes after FITC vs. PE-Cy5 fluorescence intensity plotting **(B)**. Specific profiles-based on morphological criteria were further analyzed in relation to fluorescence criteria and the specific p75NGF Receptor positive Schwann cells profiles were identified in wild-type (median of FITC = 1142; **C**), and in SOD1^G93A^ mice (median of FITC = 1305; **D**). Representative bands of PCRs for specific gene markers of Schwann cells *(S100)*, fibroblasts *(Thy1)*, and actin b (*Actb*) were searched in Schwann cells and fibroblasts enriched samples obtained by flow cytometry sorting of SOD1^G93A^ mice. Mouse whole sciatic nerve sample was used as a positive control **(E)**.

Schwann cells and fibroblasts were isolated by means of flow cytometry sorting from sciatic nerve explants of presymptomatic SOD1^G93A^ mice and their age-paired wild-type controls as described in our previous publication (Maximino et al., 2014). The sciatic nerve-derived cell suspension was submitted to a double immunolabeling to identify Schwann cells and fibroblasts by means of a fluorescein isothiocyanate (FITC)-conjugated mouse p75NGF Receptor antibody (Abcam, USA) and a fluorescein phychoerythrin (PE-Cy5)-conjugated monoclonal antibody against Thy-1 (Abcam, USA), respectively. The p75NGF Receptor labeling was employed in the cell sorting experiments because it is a well-characterized surface marker for Schwann cells (Niapour et al., 2010). Cells were then analyzed for type and specificity as well as separated on a FACSAria III Cell Sorter (BD Biosciences, USA). A maximum of 10^6^ cells were ressuspended in 500 μl of buffer. Flow cytometry dot plot Schwann cell and fibroblast profiles are shown in Figures 1A–D. Of note, the flow cytometry sorting Schwann cells of ALS mice did not show morphological differences (cell size and cytoplasmic granules) compared to control mice (Figures 1C,D). Also, the Schwann cells of ALS mice expressed high levels of mutant human SOD1, while no signal was seen in the Schwann cells of wild-type mice, as evidenced by PCR (Figure 1E).

Total RNA from enriched Schwann cells was extracted using Trizol (Life Technologies, USA) according to the manufacturer's protocol. The quantity (NanoDrop 1000 Spectrophotometer) and quality (Agilent 2100 bioanalyzer, RNA 6000 Pico LabChip) of RNAs were analyzed as described in our previous publication (Maximino et al., 2014). Also, the Schwann cell samples were submitted to PCR analyses in order to assess contamination from other cell types.

### Schwann cell enrichment and hSOD1^G93A^ verification by PCR

Schwann cells and fibroblast from sciatic nerve of 60-day-old presymptomatic SOD1^G93A^ and their wild-type controls were obtained by fluorescence activated cell sorting and submitted to PCR for sample purity verification. Total RNA from enriched Schwann cells and fibroblast was extracted using Trizol and synthesized in cDNA as described above. Primers to evaluate the presence of Schwann cells *(S100)* and fibroblast *(Thy1)* used in the PCR reactions are shown in Table 1, as well as the primer for the internal control *(Actb)*. The reactions were performed to 20 μl final volume, using GoTaq Flexi DNA Polymerase (Promega), according to the manufacturer, and 500 nM of each primer. The protocol for PCRs consisted in 95°C during 5 min, followed by 35 cycles of 95°C during 30 s, 60°C during 30 s, 72°C during 45 s, ending with 72°C in 7 min. Whole sciatic nerve sample was used as a positive control. PCR products were submitted to electrophoresis in 2% agarosis gel containing ethidium bromide for 60 min at 100 V, and then visualized under UV exposure.

**Table 1:** **Information of primers used to evaluate Schwann cell enrichment, demonstration of hSOD1^G93A^ in sciatic nerve and Schwann cell samples by PCR and to SYBR qPCR experiments in the sciatic nerve and Schwann cells isolated by means of flow cytometry sorting of 60-day-old pre-symptomatic SOD1^G93A^ mice**.

**Gene ID**	**Primer sequences (5**^**′**^**–3**^**′**^**)**		**Amplicon (BP)**
*S100*	F: CCCTCATTGATGTCTTCCACC	R: TCTCCATCACTTTGTCCACC	150
*Thy1*	F: GTCCTTACCCTAGCCAACTTC	R: CCGCCACACTTGACCAG	134
*hSOD1^G93A^*	F: ATCAGCCCTAATCCATCTGA	R: CGCGACTAACAATCAAAGTGA	236
*Ngfr*	F: CCTCATTCCTGTCTATTGCTCC	R: TGGCTCCTTGTTTATTTTGCTTG	107
*H2afx*	F: TTGTTCGCAGCTCTTCTACC	R: GTAGTGGCCTTTCCGCAG	149
*Mapk10*	F: TGTTAGTGATTGACCCAGCG	R: TGTGCTCCCTTTCATCCAG	141
*Cdkn1a*	F: CAGATCCACAGCGATATCCAG	R: AGAGACAACGGCACACTTTG	103
*Cdkn1b*	F: TGGACCAAATGCCTGACTC	R: GGGAACCGTCTGAAACATTTTC	144
*E2f1*	F: TCTCTTTGACTGTGACTTTGGG	R: TCGTGCTATTCCAATGAGGC	147
*Traf2*	F: ACTTCACCAGAAAGCGTCAG	R: GGTTTTCTCTGTAGGTCTTCCG	148
*Foxo3*	F: CGTTGTTGGTTTGAATGTGGG	R: TGCATCACTCGTTCATCCTG	143
*Mtor*	F: ATTCAATCCATAGCCCCGTC	R: ACAGTTCCAAAGACACCAGAG	143
*Erbb3*	F: GGGCTATGAGACGCTACTTG	R: TGCAGGACAAACTAAGGAGTG	145
*Hspa1a*	F: TCGAGGAGGTGGATTAGAGG	R: TGCAGGACAAACTAAGGAGTG	120
*Prdx2*	F: CCCTGAATATCCCTCTGCTTG	R: TTGACTGTGATCTGGCGAAG	139
**NORMALYZER**			
*Actb*	F: ACCTTCTACAATGAGCTGCG	R: CTGGATGGCTACGTACATGG	147

*S100 (S100 calcium binding protein), Thy1 (THYmocyte differentiation antigen 1), hSOD1^G93A^ (mutated human superoxide dismutase 1), Ngfr (Nerve growth factor receptor), H2afx (H2A histone family, member X), Mapk10 (mitogen-activated protein kinase 10), Cdkn1a (cyclin-dependent kinase inhibitor 1A (P21)), Cdkn1b (cyclin-dependent kinase inhibitor 1B), E2f1 (E2F transcription factor 1), Traf2 (TNF receptor-associated factor 2), Foxo3 (forkhead box O3), Mtor (mechanistic target of rapamycin-serine/threonine kinase), Erbb3 (v-erb-b2 erythroblastic leukemia viral oncogene homolog 3-avian), Hspa1a (heat shock protein 1A), Prdx2 (peroxiredoxin 2) and Actb (Actin, Beta)*.

Sciatic nerve and Schwann cells of 60-day-old presymptomatic SOD1^G93A^ and their wild-type controls were submitted to PCR for verification the presence of human SOD1^G93A^ (hSOD1^G93A^). The procedures were the same as described above. Primers to evaluate the presence of *hSOD1*^G93A^ and *Actb* (internal control) used in the PCR reactions are shown in Table 1.

### Quantitative PCR

A subset of genes was chosen for verification of expression patterns based on their possible involvement in ALS/neurodegeneration-related death, stress and mitochondrion or based on their higher level of connectivity (betweenness/degree). qPCR was performed on samples from mouse sciatic nerves and also enriched Schwann cells as previously described (Maximino et al., 2014). Briefly, cDNA was synthesized from 100 ng of total RNA using the Maxima First Strand cDNA Synthesis Kit (Thermo Scientific, USA), according to manufacturer's instructions. qPCR reactions were carried out in duplicate with 10 ng cDNA, using the DyNAmo ColorFlash SYBR Green qPCR kit (Thermo Scientific, USA) and 400 nM of each primer in a final reaction volume of 20 μl. Reactions were run with the Applied Biosystems 7500 Real-Time PCR System (Applied Biosystems). Sequence information regarding the SYBR primers can be found in Table 1. qPCR of *Foxo3* was also performed on enriched fibroblasts obtained from the mouse sciatic nerves by means of flow cytometry cell sorting (Figure S1).

Thermocycling conditions for SYBR reactions included an initial denaturation at 95°C for 10 min. Templates were amplified for 40 cycles at 95°C for 15 s, and for additional 40 cycles for at 60°C for 30 s. A dissociation curve was then generated to ensure amplification of a single product, and the verification of no primer dimer formation. A standard curve was generated for each primer pair in order to determine the efficiency of the PCR reaction over a range of template concentrations from 0.032 to 20 ng/μl, using cDNA synthesized from reference mouse RNA. The efficiency for each set of primers was 100 ± 5%. Gene expression was normalized to the expression of *Actb* and determined using the ΔΔCt mathematical model (Livak and Schmittgen, 2001). *Actb* was chosen as a housekeeping gene to normalize the qPCR values because the microarray analysis showed no alteration in expression of this gene across samples.

### Statistical analysis

The statistical method employed for the microarray analysis is described in details in the microarray analysis section. Furthermore, a two-tailed unpaired *t*-test was used to evaluate the level of significance of gene expression independently between the two genotypes (SOD1^G93A^ × wild-type) in the qPCR analyses. Analyses were performed using the GraphPad Prism 5 (San Diego, CA). Data were presented as Means ± Standard Error of Mean (SEM) and significance level was set to *p* < 0.05.

## Results

### Qualitative analyses of histological sections and enriched schwann cells of the sciatic nerves

**Figure 2:**
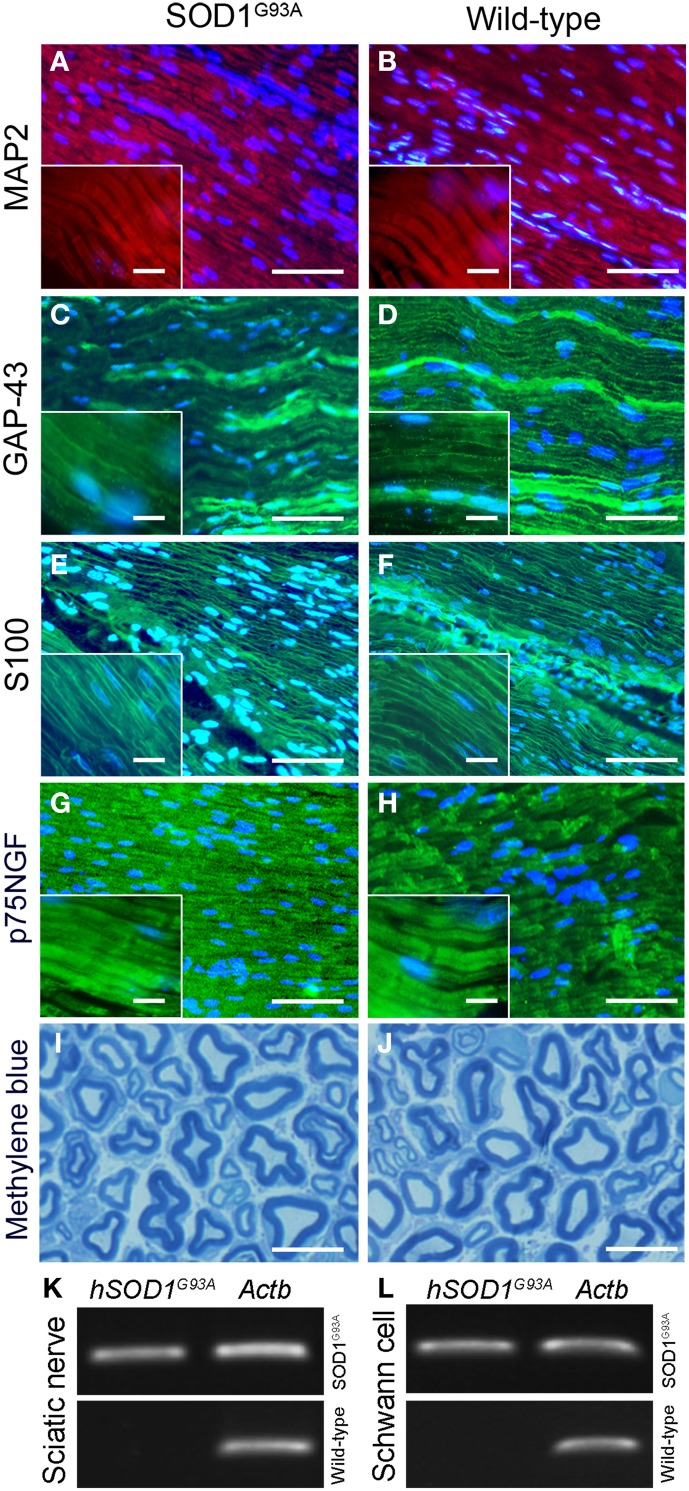
**Histopathological analysis (A–J) and molecular evaluation of ***hSOD1***^G93A^ signal (B) in the sciatic nerve and Schwann cell samples of 60-day-old presymptomatic SOD1^G93A^ and aged paired wild-type mice**. Immunofluorescence staining of MAP2 (**A,B**; red), GAP-43 (**C,D**; green), S100 (**E,F**; green) and p75NGF (**G,H**; green) in the sciatic nerve of 60-day-old presymptomatic SOD1^G93A^ mice **(B,D,F,H)** and their wild-type controls **(A,C,E,G)**. MAP2 and GAP-43 are markers of neuronal fibers; S100 and p75NGF are markers of Schwann cells. Cell nuclei were stained with DAPI (blue). The insert boxes in the bottom left of images show a higher magnification of the cell profiles. Methylene blue staining of Schwann cell myelin sheets of sciatic nerve of 60-day-old presymptomatic SOD1^G93A^ mice **(J)** and their wild-type controls **(I)** are also seen. Scale bars: 10 μm. Of note, the same staining pattern was observed for both genotypes (SOD1^G93A^ and wild-type controls) for all cell markers and for the histological sections. Representative bands of PCR for specific gene markers of human SOD1^G93A^ (*hSOD1*^G93A^) and actin b (*Actb*) in sciatic nerve **(K)** and Schwann cells enriched samples **(L)** obtained by flow cytometry sorting of SOD1^G93A^ and wild-type control mice.

No qualitative changes were found regarding morphology of sciatic nerves of presymptomatic ALS mice compared to control at histopathological examination (Figures 2A–J). PCR analysis of sciatic nerve and Schwann cells enriched by flow cytometry showed the presence of hSOD1G93A in the SOD1G93A mice, but not in the wild-type controls (Figures 2K,L). Also, the flow cytometry sorting Schwann cells of ALS mice did not show morphological differences (cell size and cytoplasmic granules) compared to control mice (Figures 1C,D).

### Verification of microarray results by quantitative PCR

The results of qPCR verification of 10 representative genes in the sciatic nerve of presymptomatic SOD1^G93A^ mice are shown in Table 2. The up and down-regulations of the verified genes in the sciatic nerve of 60-day-old SOD1^G93A^ mice by means of qPCR were coincident and supported the microarray findings of correspondent animal ages (Table S1).

**Table 2:** **qPCR data of selected genes for verification in the sciatic nerve of 60-day-old pre-symptomatic SOD1^G93A^ mice**.

**Gene symbol**	**Fold change**	***p*-value**
*Ngfr*	1.88	0.0051
*E2f1*	1.41	0.0353
*Traf2*	1.37	0.0150
*Erbb3*	1.29	0.0390
*Cdkn1a*	1.26	0.0297
*Foxo3*	−1.96	0.0023
*Hspa1a*	2.06	0.0006
*Prdx2*	1.51	0.0359
*Mapk10*	1.35	0.0442
*Mtor*	−1.23	0.0443

*qPCR of differentially expressed genes in sciatic nerves of SOD1^G93A^ mice compared to the wild-type controls selected from microarray data (p < 0.05; see text for details). The genes are related to death, stress and mitochondrion. The means of fold change and p-values are shown, according unpaired two-tailed t-test. The regulation of studied genes for verification showed the same direction as that found in the microarray experiments (Table 3)*.

### Bioinformatics analysis

#### Functional enrichment analysis

The DAVID analysis of differentially expressed genes in the sciatic nerve of 60-day-old SOD1^G93A^ mice revealed 19 GO terms of Biological Process related to cell death and apoptosis (genes related to Death in Table 3). These GO terms of genes related to Death showed 112 dysregulated genes (46 down and 66 up-regulated genes). Furthermore, DAVID also identified two GO terms of Biological Process related to Stress (Table 3). These GO terms related to Stress showed 66 dysregulated genes (31 down and 35 up-regulated genes). Finally, DAVID identified two GO terms of Cellular Components related to mitochondrial function (Table 3). These GO terms of Mitochondrion showed 143 dysregulated genes (91 down and 52 up-regulated genes). Table 3 shows the down regulated and up regulated genes of Death, Stress, Mitochondrion categories with fold change equal or higher than 1.5. The deregulated genes of these categories with fold change smaller than that are shown in the Table S2 of the Supplementary Material. We have not expected to find gene regulation with high degree of fold change in this stage of presymptomatic events, a period in which dramatic occurrences related to inflammation, neurodegeneration and necrosis are not taking place. The above-mentioned cut-off in the Table 3 is just to facilitate the demonstration of the relatively higher deregulated genes. All analyses of this study were performed without a cut-off.

**Table 3:** **Differentially expressed genes related to death, stress and mitochondrion in the sciatic nerve of 60-day-old SOD1^G93A^ mice**.

**Genes related to death**						**Genes related to stress**						**Genes related to mitochondrion**					
**Down regulated**			**Up regulated**			**Down regulated**			**Up regulated**			**Down regulated**			**Up regulated**		
**Gene**	**Fold**	**References**	**Gene**	**Fold**	**References**	**Gene**	**Fold**	**References**	**Gene**	**Fold**	**References**	**Gene**	**Fold**	**References**	**Gene**	**Fold**	**References**
*Htatip2*	−7.32		*Fem1b*	1.26		*Pttg1*	−3.44		*Nhej1*	1.26		*Acsm3*	−6.15		*Prdx2*	1.26	
*Tcf15*	−2.54		*Prdx2*	1.26		*Obfc2a*	−3.02		*Prdx2*	1.26		*Isoc2a*	−3.02		*Ctsa*	1.28	
*Pax2*	−2.39		*E2f1*	1.28	[10]	*Fancc*	−2.69		*Mre11a*	1.28		*Acss3*	−2.82		*Nme6*	1.28	
*Asah2*	−2.22		*Apoe*	1.29	[11]	*Fam175a*	−2.32		*H2afx*	1.29		*Dact2*	−2.49		*Acly*	1.30	
*Vnn1*	−2.10		*Traf2*	1.30		*Txnip*	−2.25		*Apoe*	1.29	[11]	*Mgst1*	−2.47		*Cox4nb*	1.30	
*Rrm2b*	−2.01		*Cd24a*	1.31		*Polg2*	−2.03		*Mdfic*	1.30		*Rsad1*	−2.42		*Trmt2b*	1.30	
*Tnfsf10*	−2.01		*Traf4*	1.32		*Rrm2b*	−2.01		*Ppp1r15b*	1.32		*Asah2*	−2.22		*Ctsb*	1.31	
*Gdf5*	−2.01		*Pura*	1.33		*Cln3*	−1.81		*Hipk2*	1.33		*Akap1*	−2.21		*Synj2bp*	1.36	
*Dapl1*	−2.00		*Ccar1*	1.33		*Epas1*	−1.78		*Cdkn1a*	1.34	[12,13]	*Cyb5r2*	−2.19		*Slc25a38*	1.37	
*Thoc1*	−1.95		*Hipk2*	1.33		*Nudt15*	−1.72		*Timeless*	1.37		*Dnm3*	−2.18		*Tmem65*	1.37	
*Trib3*	−1.87		*Shf*	1.34		*Fancm*	−1.55		*Brip1*	1.38		*Adhfe1*	−2.13		*Chchd4*	1.37	
*Cln3*	−1.81		*Cdkn1a*	1.34	[12,13]	*Cygb*	−1.49		*Gadd45a*	1.40		*Prr5l*	−2.04		*Opa3*	1.40	
*Tgfb2*	−1.80	[1,2,3]	*Rhob*	1.35		*Tipin*	−1.48		*Ctsd*	1.41		*Polg2*	−2.03		*Ctsd*	1.41	
*Bnip3*	−1.76		*Bcl6*	1.35		*Cry2*	−1.48		*Hspa5*	1.44		*Rrm2b*	−2.01		*Slc25a15*	1.42	
*Gzmc*	−1.73		*Ascl1*	1.35		*Rad51l3*	−1.44		*Hipk1*	1.46		*Slc25a30*	−2.01		*Tomm20l*	1.43	
*Osm*	−1.69		*Plagl2*	1.36		*Xpc*	−1.42		*Oxsr1*	1.51		*Nudt19*	−1.99		*Slc25a25*	1.43	
*Rhot1*	−1.68		*Rabep1*	1.37		*Eif2ak2*	−1.42	[4]	*Sfpq*	1.51		*Qtrtd1*	−1.97		*Alkbh7*	1.44	
*Aifm2*	−1.65		*Alms1*	1.37		*Xpa*	−1.39		*Neil3*	1.52		*Gtpbp10*	−1.93		*Tomm40l*	1.46	
*Twist1*	−1.62		*Alox12*	1.39		*Pnkp*	−1.38		*Slk*	1.63	[17]	*Adh1*	−1.89		*Cerk*	1.50	
*Trp53inp1*	−1.57		*Bdkrb2*	1.43		*Xrn2*	−1.38		*Uhrf1*	1.79		*Mtch2*	−1.88		*Bcat2*	1.54	
*Dffa*	−1.56		*Sphk2*	1.44		*Aplf*	−1.37		*Cep63*	1.88		*Acat3*	−1.87		*Gramd4*	1.61	
*Foxl2*	−1.54		*Acin1*	1.46		*Eif2ak4*	−1.32		*Mapk10*	1.98		*Pecr*	−1.86		*Gm13570*	1.61	
*Traf1*	−1.51		*Hipk1*	1.46		*Cat*	−1.31		*Trpc2*	2.02		*Isoc2b*	−1.82		*Cox16*	1.64	
*Gatad2a*	−1.50		*Lst1*	1.47		*Gclc*	−1.29		*Bcl3*	2.08		*Chchd7*	−1.81		*Aco1*	1.64	
*Foxc2*	−1.45		*Skp2*	1.47		*Foxo3*	−1.27	[8,9]	*Hspa1a*	2.19		*Acaca*	−1.79		*Dusp18*	1.66	
*Xpa*	−1.43		*Dnase1*	1.48		*Rad52*	−1.27		*Bard1*	2.29		*Gadd45gip1*	−1.78		*Itga10*	1.73	
*Pigt*	−1.43		*Dcc*	1.55		*Esco1*	−1.26		*Rad23a*	2.37		*Bnip3*	−1.76		*Tstd1*	1.74	
*Klk8*	−1.42		*Ngfr*	1.56	[14,15]				*Btg2*	2.46		*Alas1*	−1.75		*Abcd1*	1.76	
*Eif2ak2*	−1.42	[4]	*Phlda1*	1.58		**GO**		**Term**				*Mapk12*	−1.74		*Mthfd2*	1.88	
*Zfp346*	−1.41		*Cdkn1b*	1.59	[16]	GO:0033554		Cellular response to stress				*Acacb*	−1.72		*Mapk10*	1.98	
*Gli3*	−1.40	[5]	*Gramd4*	1.61		GO:0006979		Response to oxidative stress				*Tfam*	−1.70	[23,24]	*Rnaset2a*	2.08	
*Cx3cl1*	−1.40	[6]	*Slk*	1.63	[17]							*Cds2*	−1.69		*Hspa1a*	2.19	
*Ripk3*	−1.37		*Bag5*	1.68								*Rhot1*	−1.68		*Pak7*	2.41	
*Apbb3*	−1.36		*Fcgr1*	1.69								*Aifm2*	−1.65				
*Bmp4*	−1.34		*Ripk2*	1.70								*Maob*	−1.62				
*Mtap1s*	−1.34		*Nrp*	1.70								*Mrps2*	−1.62				
*Htra2*	−1.30	[7]	*Gzmm*	1.74								*Mars2*	−1.61				
*Gclc*	−1.29		*Timp1*	1.78								*Cpt1a*	−1.61				
*Foxo3*	−1.27	[8,9]	*Sphk1*	1.93								*Sfxn1*	−1.61				
			*Tnfrsf12a*	1.96								*Ugt1a10*	−1.60				
			*Cx3cr1*	2.05	[6, 18, 19]							*Herc2*	−1.57	[25,26,27]			
			*Bcl3*	2.08								*Mtor*	−1.57				
			*Hspa1a*	2.19								*Gstz1*	−1.56				
			*Bard1*	2.29								*Ehhadh*	−1.53				
			*Pou4f1*	2.30								*Acad8*	−1.52				
			*Pak7*	2.41								*Mrps10*	−1.51				
			*Btg2*	2.46								*Aldh18a1*	−1.48				
			*Erbb3*	2.74	[20]							*Gprc5c*	−1.48				
			*Shh*	3.50	[5,21]							*Echdc2*	−1.48				
			*Gjb6*	3.79								*Mlxip*	−1.47				
			*Gdnf*	11.18	[22]							*Nipsnap3b*	−1.47				
**GO**		**Term**										*Hmgcs2*	−1.47				
GO:0010941		Regulation of cell death										*Ociad1*	−1.45				
GO:0043067		Regulation of programmed cell death										*Agxt2l2*	−1.43				
GO:0042981		Regulation of apoptosis										*Suclg2*	−1.40				
GO:0016265		Death										*Nd2*	−1.40				
GO:0008219		Cell death										*Ass1*	−1.40				
GO:0012501		Programmed cell death										*Mrpl9*	−1.40				
GO:0006915		Apoptosis										*Nd1*	−1.39				
GO:0043069		Negative regulation of programmed cell death										*Fitm2*	−1.39				
GO:0060548		Negative regulation of cell death										*Decr1*	−1.38				
GO:0043066		Negative regulation of apoptosis										*Aldh6a1*	−1.36				
GO:0010942		Positive regulation of cell death										*Bbox1*	−1.35				
GO:0043068		Positive regulation of programmed cell death										*Etfa*	−1.35				
												*Sirt5*	−1.33	[28]			
												*Osgepl1*	−1.32				
												*Msra*	−1.31				
												*Cat*	−1.31	[29,30]			
												*Supv3l1*	−1.30				
												*Htra2*	−1.30	[7]			
												*Nme4*	−1.29				
												*Nfs1*	−1.29				
												*Nlrx1*	−1.28				
												*Lactb2*	−1.27				
												*Parl*	−1.26				
												**GO**		**Term**			
												GO:0005739		Mitochondrion			
												GO:0005741		Mitochondrial outer membrane			

*Enriched analysis by means DAVID tool pointed genes from Gene Ontology (GO) Biological Processes terms related to Death and Stress, and Cell Components related to Mitochondrion. EASE score was set to 0.05. Positive and negative values pointed to down and upregulated genes expressions, respectively. The genes with fold between -1.25 and 1.25 are shown in the Supplementary Material (Table S2). References report to previous publications of respective genes or their related gene products in the context of Amyotrophic Lateral Sclerosis: 1, Day et al., 2005; 2, Katsuno et al., 2011; 3, Murakami et al., 1999; 4, de Oliveira et al., 2014; 5, Peterson and Turnbull, 2012; 6, Sun et al., 2013; 7, Kawamoto et al., 2010; 8, Léger et al., 2006; 9, Mojsilovic-Petrovic et al., 2009; 10, Ranganathan and Bowser, 2003; 11, Haasdijk et al., 2002; 12, Gonzalez de Aguilar et al., 2008; 13, Manzano et al., 2013; 14, Kerkhoff et al., 1991; 15, Oliveira et al., 1994; 16, Cova et al., 2010; 17, Kudo et al., 2010; 18, Beers et al., 2011;19, Lopez-Lopez et al., 2014; 20, Gorlewicz et al., 2009; 21, Ma et al., 2013; 22, Manabe et al., 2003; 23, Morimoto et al., 2012; 24, Thau et al., 2012; 25, Morimoto et al., 2007; 26, Yang et al., 2001; 27, Zhang et al., 2011; 28, Körner et al., 2013; 29, Reinholz et al., 1999; 30, Nikolic-Kokic et al., 2006*.

**Figure 3:**
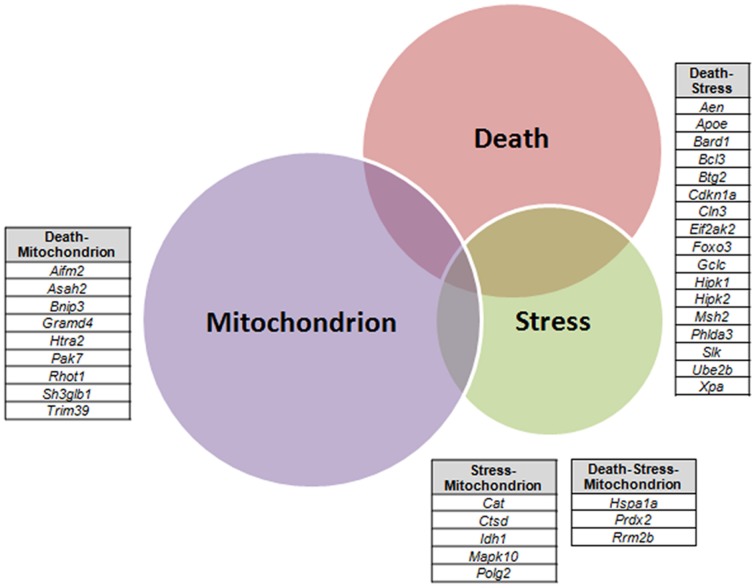
**Venn diagram of differentially expressed genes related to Death, Stress and Mitochondrion categories in sciatic nerve (60-day-old) of SOD1^G93A^ animals compared to wild-type controls by means of microarray experiments**. The lists of genes of the enriched categories were obtained by means of DAVID tool based on Biological Process and Cellular Component Ontology (EASE score set to 0.05), which identified 112 differentially expressed genes in Death, 66 genes in Stress, and 143 genes in Mitochondrion categories. Venn diagram demonstrates genes which are common to Death and Stress (17 genes), Death and Mitochondrion (nine genes), and also Stress and Mitochondrion (nine genes). Three genes (*Hspa1a, Prdx2, Rrm2b*) were present in the three categories (Death, Stress, and Mitochondrion).

A VEEN diagram of those dysregulated genes related to Death and Stress and Mitochondrion showed 17 genes belonging to both Death and Stress groups of genes, 9 genes belonging to both Death and Mitochondrion groups of genes, 5 genes belonging to both Stress and Mitochondrion groups of genes, and finally 3 genes belonging to the three, Death, Stress, and Mitochondrion groups of genes (Figure 3).

**Figure 4:**
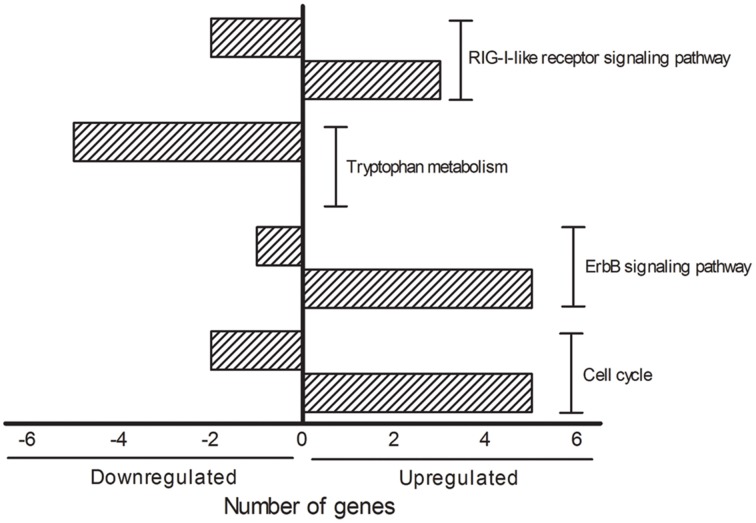
**KEGG pathways showing the number of transcripts up (right side bars with positive values) and down-regulated (left side bars with negative values)**. The figure is representative of the KEGG pathways (EASE score was set to 0.05) obtained from the GO terms list containing genes related to death, stress and mitochondrion that have been already mentioned in the ALS literature. The categories were composed by seven genes (Cell cycle), six genes (ErbB signaling pathway), five genes (Tryptophan metabolism and RIG-I-like receptor signaling pathway).

The DAVID analysis of dysregulated genes related to Death, Stress and Mitochondrion shown in Table 3 are identified with 16 KEGG pathways (Table 4). Furthermore, those identified KEGG pathways that have been previously described in the literature as being related to ALS include RIG-I-like receptor signaling (2 down and 3 up-regulated genes; Figure 4), tryptophan metabolism (5 down-regulated genes; Figure 4), ErbB signaling (1 down and 5 up-regulated genes; Figure 4) and cell cycle (2 down and 5 up-regulated genes; Figure 4). Of note, Cancer, Small cell lung cancer, the Adipocytokine signaling pathway and Chronic myeloid leukemia pathways are likely not related to ALS and were not included in Table 4.

**Table 4:** **KEGG pathways obtained from gene lists related to Death, Stress and Mitochondrion from 60-day-old SOD1^G93A^ sciatic nerve microarray analyses**.

**Term**	**Pathways**	**Number of genes**	**Categories/Genes**		
			**Death**	**Stress**	**Mitochondrion**
mmu00280	Valine, leucine and isoleucine degradation	12			*Abat, Acaa2, Acad8, Acadsb, Acat1, Aldh6a1, Aldh9a1, Bcat2, Bckdha, Ehhadh, Hmgcs2, Oxct1*
mmu00640	Propanoate metabolism	8			*Abat, Acaca, Acacb, Acat1, Aldh6a1, Aldh9a1, Ehhadh, Suclg2*
mmu00650	Butanoate metabolism	7			*Abat, Acat1, Acsm3, Aldh9a1, Ehhadh, Hmgcs2, Oxct1*
mmu00071	Fatty acid metabolism	7			*Acaa2, Acadsb, Acat1, Adh1, Aldh9a1, Cpt1a, Ehhadh*
mmu04110	Cell cycle	7	*Cdkn1a, Cdkn1b, E2f1, Skp2, Tgfb2*	*Gadd45a, Pttg1, Cdkn1a*	
mmu04012	ErbB signaling pathway	6	*Cdkn1a, Cdkn1b, Erbb3, Pak7*	*Mapk10, Cdkn1a*	*Pak7, Mapk10, Mtor*
mmu00020	Citrate cycle (TCA cycle)	5			*Aco1, Acly, Idh1, Idh3a, Suclg2*
mmu00380	Tryptophan metabolism	5		*Cat*	*Acat1, Aldh9a1, Cat, Ehhadh, Maob*
mmu00480	Glutathione metabolism	5	*Gclc, Rrm2b*	*Gclc, Rrm2b*	*Gstz1, Idh1, Mgst1, Rrm2b*
mmu00330	Arginine and proline metabolism	5			*Aldh18a1, Aldh9a1, Ass1, Glud1, Maob*
mmu04622	RIG-I-like receptor signaling pathway	5	*Traf2, Traf3*	*Mapk10*	*Mapk10, Mapk12, Nlrx1*
mmu00982	Drug metabolism	5			*Adh1, Gstz1, Maob, Mgst1, Ugt1a10*
mmu00310	Lysine degradation	4			*Acat1, Aldh9a1, Bbox1, Ehhadh*
mmu00620	Pyruvate metabolism	4			*Acaca, Acacb, Acat1, Aldh9a1*
mmu00600	Sphingolipid metabolism	4	*Sphk1, Sphk2*		*Asah2, Cerk*
mmu00072	Synthesis and degradation of ketone bodies	3			*Acat1, Hmgcs2, Oxct1*

*KEGG pathways were obtained from gene lists related to Death, Stress and Mitochondrion from 60-day-old SOD1^G93A^ sciatic nerve microarray analyses and genes included in these pathways and categories are shown in the table. EASE score was set to 0.05*.

#### Protein interaction network analysis from dysregulated genes

**Figure 5:**
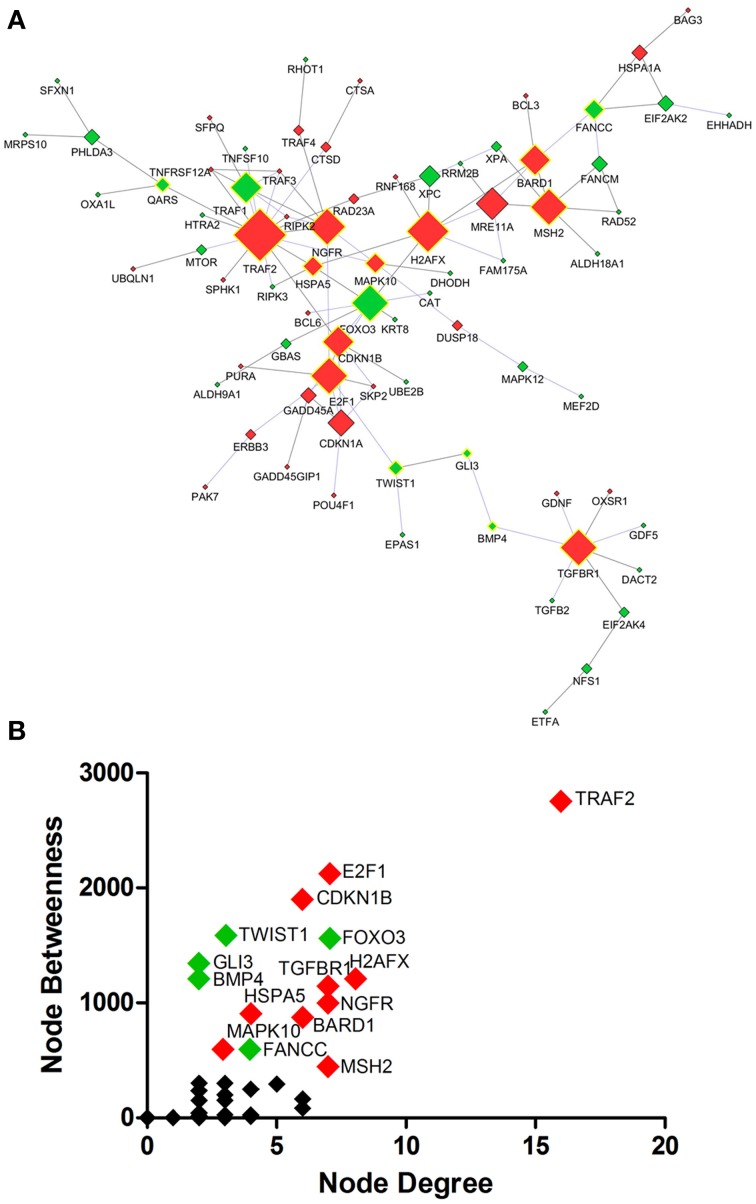
**Protein interaction network developed from differentially expressed genes related to Death, Stress and Mitochondrion of sciatic nerves from SOD1^G93A^ mice (A)**. Scatter plot of the correlation between highest values of node degree (hubs) and node betweenness (bottlenecks) is shown in **(B)**, as described in the text. Up and down-regulated genes are represented respectively as red and green diamonds. Nodes with the highest values for node degree (number of connections) and node betweenness (number of shortest paths) are represented with a yellow border. Of note from this analysis, the genes *E2f1, Foxo3, Gli3, Ngfr, Cdkn1a* or their related products were already described in the context of ALS.

Protein interaction network analysis using dysregulated genes related to Death, Stress and Mitochondrion showed the hubs (Figure 5A) TRAF2 (16 connectors), H2AFX (8 connectors) and E2F1, FOXO3, MSH2, NGFR, TGFBR1 (7 connectors). Furthermore, the network analysis showed five bottlenecks (TRAF2, E2F1, CDKN1B, TWIST1, and FOXO3). The scatter plot of the values of the hubs node degree vs. the values of node betweenness is shown in Figure 5B. Of note, TRAF2, E2F1, CDKN1B, FOXO3, and H2AFX occupied the highest positions in the scatter plot (Figure 5B). Networks and scatter plots produced from the analyses of the specific genes of Death, Stress and Mitochondrion categories were shown in Figures S2–S5 of Supplementary Material.

### Schwann cell and qPCR experiments

**Figure 6:**
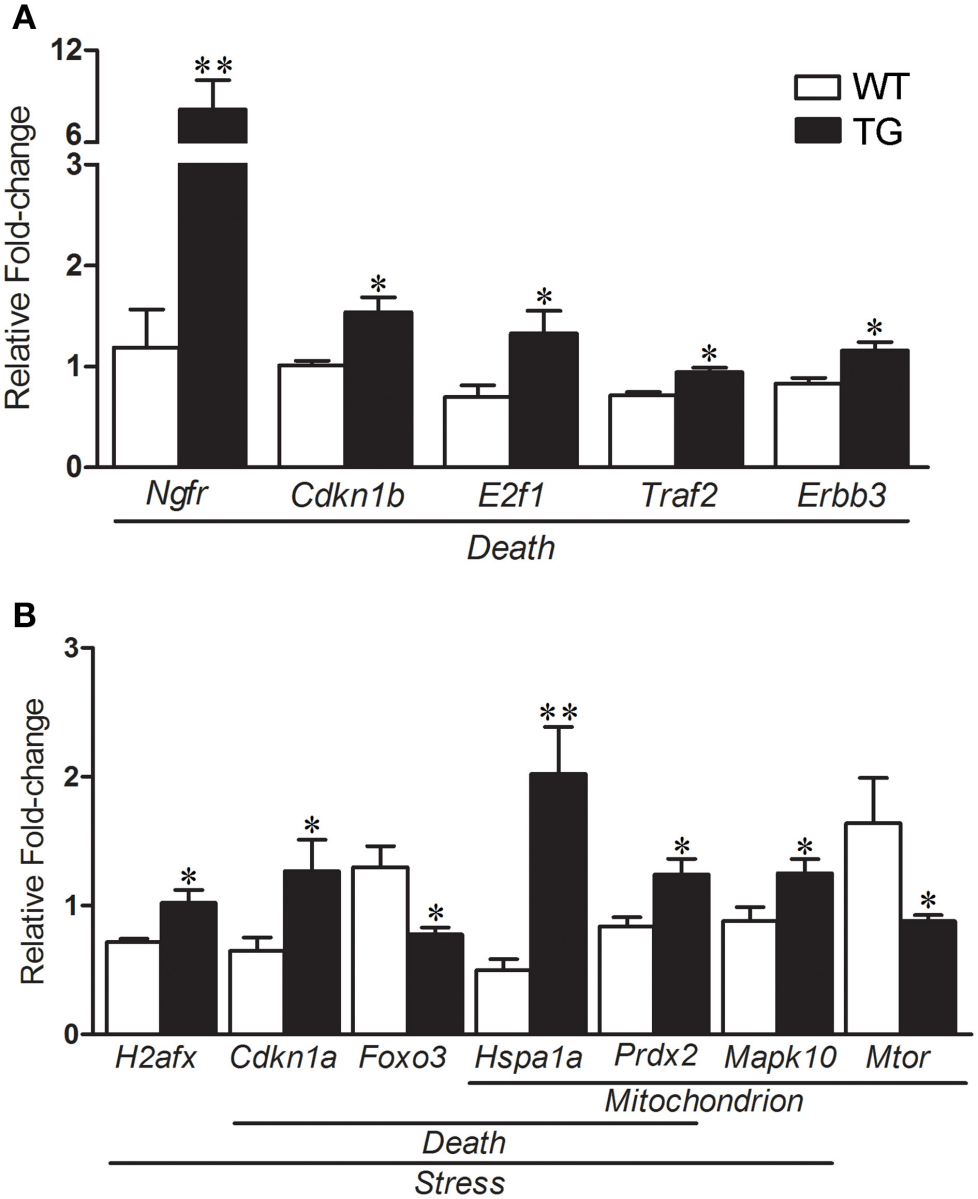
**Relative fold change values of selected dysregulated genes related to death, stress and mitochondrion by qPCR in enriched sciatic nerve-derived Schwann cell samples of 60-day-old SOD1^G93A^ (TG) mice compared to the age matched wild-type (WT) mice**. Schwann cells were enriched by means of flow cytometry cell sorting technique from the sciatic nerves of the mice. Significant increases in expression are seen in genes related to Death (*Ngfr, Cdkn1b, E2f1, Traf2*, and *Erbb3;* **A**). Up-regulated *H2afx* is related to Stress category **(B)**. Down-regulated *Cdkn1a* and *Foxo3* are related to Death and Stress categories respectively **(B)**. Up-regulated *Hspa1* and *Prdx* belonged to Death, Stress and Mitochondrion categories **(B)**. Up-regulated *Mapk10* (Stress and Mitochondrion categories) and down-regulated *Mtor* (Mitochondrion) were also shown down-regulated **(B)**. A pool of the non-ALS group was used as reference samples with a reference value of 1, see text for details. Means ± SEM; *n* = 6 for each group; ^*^ and ^**^p-values indicates the < 0.05 and < 0.01 levels of significance, respectively, according to unpaired two-tailed *t*-test.

The results of selected genes for verification in the mouse sciatic nerves by qPCR are shown in the Table 2. qPCR analyses of gene expression from the enriched Schwann cells isolated from sciatic nerves of presymptomatic 60-day-old SOD1^G93A^ mice identified a number of up-regulated genes including *Ngfr* (6.85-fold)*, Cdkn1b* (1.51-fold)*, E2f1* (1.89-fold)*, Traf2* (1.31-fold) and *Erbb3* (1.38-fold) related to Death (Figure 6A). The gene *H2afx* (1.42-fold) of the Stress group was up-regulated. The genes *Cdkn1a* (1.95-fold) and *Foxo3* (-1.67-fold) from the Death and Stress groups were up- and down regulated, respectively. The genes *Hspa1a* (4.48-fold) and *Prdx2* (1.48-fold) of the Death, Stress and Mitochondrion groups were up-regulated. *Mapk10* (1.42-fold) of the Stress and Mitochondrion groups was up-regulated and *Mtor* (−1.86-fold), related to mitochondrion, was down regulated (Figure 6B). Interestingly, the expression of *Foxo3* was not altered in the enriched fibroblasts of the mouse sciatic nerve (Figure S1).

## Discussion

### Triggering mechanisms of motor neuron death in ALS

Several mechanisms have been proposed as triggers for both autonomous and non-autonomous events related to motor neuron death in ALS. Events of oxidative stress, neuroimmune reactions, protein aggregation, glutamate excitotoxicity, mitochondrial dysfunction and impaired axonal transport in ALS are currently under investigation (Bruijn et al., 2004; Boillée et al., 2006a; de Vos et al., 2007; Jaiswal and Keller, 2009; Redler and Dokholyan, 2012).

The initial triggering and the secondary reactive events associated with motor neurons and their neighboring glial cells are unknown (Tapia, 2014). For instance, early findings related to morpho/physiological changes have been described in presymptomatic phases of the ALS mouse model (Boillée et al., 2006a; Ferraiuolo et al., 2007; Alves et al., 2011; de Oliveira et al., 2013, 2014; Maximino et al., 2014). Of note, fragmentation of the Golgi apparatus (Mourelatos et al., 1996), vacuolization of mitochondria (Bendotti et al., 2001), deficits in axonal transport (Ikenaka et al., 2012), endoplasmic reticulum stress (Tadic et al., 2014), the activation of glial cells (microglia and astrocytes) (Nagai et al., 2007; Graber et al., 2010) and electrophysiological changes (Quinlan, 2011) have all been described within motor neurons and neighboring cells at early postnatal ages. Technological advances have facilitated the discovery of molecular events underlying both autonomous (Wada et al., 2012) and non-autonomous (Ferraiuolo, 2014) mechanisms possibly related to early presymptomatic neuronal toxicity in ALS (Arbour et al., 2015; Saba et al., 2015).

Evidence indicates the existence of very early events, possibly anticipating the motor neuron death, that take place peripherally, i.e., in the motor nerve, motor nerve terminals, neuromuscular junction and muscle (Rocha et al., 2013; de Oliveira et al., 2014; Moloney et al., 2014). In fact, very early electrophysiological events in the motor nerve precede cell body disappearance in the spinal cord as well as the onset of clinical symptoms in ALS (Alves et al., 2011). Absence of neuronal death or morphological alterations in the motor neuron cell bodies has been described in the 60-days-old presymptomatic SOD1 mice (Fischer et al., 2004; Gould et al., 2006; Casas et al., 2013).

Remarkably, early morphological, biochemical and molecular changes in peripheral non-neuronal cells, i.e., Schwann cells and skeletal muscles, are emerging as dying-back mechanisms of motor neuron degeneration in ALS (de Winter et al., 2006; Dupuis and Loeffler, 2009; Dupuis et al., 2009; Keller et al., 2009; Narai et al., 2009; Chen et al., 2010; Dadon-Nachum et al., 2011; Venkova et al., 2014). In fact, the involvement of Schwann cells, which maintain close morphological/physiological relationships with motor axons, has gained more attention in ALS (de Winter et al., 2006; Keller et al., 2009; Chen et al., 2010; Maximino et al., 2014; Venkova et al., 2014). Our work has contributed to a more detailed understanding of the mechanisms involved in the dying-back events associated with motor nerves in ALS by performing a large-scale gene profiling analysis utilizing a microarray analyses in the sciatic nerve of presymptomatic ALS mice.

A deficit in the paracrine trophic interactions between Schwann cells and motor neurons in presymptomatic ALS is a logical choice for further study since Schwann cells offer the main source of trophic stimuli for the maintenance of mature motor neurons (Bhatheja and Field, 2006) and for the regeneration of their fibers after injury (Gupta et al., 2005). In support of this hypothesis, mSOD1 gene expression in distal Schwann cells has been suggested to interfere with the trophic maintenance of motor axon projections (Inoue et al., 2003). Also, reduction of Schwann cell-derived insulin growth factor-1 and cilliary neurotrophic factor in sciatic nerves of both ALS mice and patients have been correlated to disease worsening (Lee et al., 1996; Lobsiger et al., 2009).

In addition to a deficit in paracrine trophic factor maintenance, the presence of toxic factors from Schwann cells affecting motor neurons at early presymptomatic stages in ALS is an additional matter for investigation. Indeed, our DAVID analyses of dysregulated gene expression in sciatic nerves of presymptomatic SOD1^G93A^ mouse model highlighted the GO categories death, stress and mitochondrion, which may be related to the above mentioned possibilities. Of note, the findings of a high number of dysregulated genes related to death, with many also included in the stress and mitochondrial function categories, indicated a complex regulation of these events before motor neuron death, as described for other neurodegenerative disorders (Friedman et al., 1996; Gatzinsky et al., 2003; Pun et al., 2006; Campana, 2007; Lobsiger et al., 2009; Nobbio et al., 2009a,b). Conversely, negative and positive gene regulation related to death and anti-apoptosis mechanisms were also identified in several categories of dysregulated genes, indicating a concomitant regulation of cell toxicity/maintenance before neurodegeneration during the presymptomatic stages in ALS. It should be emphasized that the present description of gene profiling in the sciatic nerve in the presymptomatic ALS mouse model may represent predominantly the regulation of Schwann cells transcripts. In fact, as discussed below, the qPCR verification of selected genes in the mouse sciatic nerve tissue has confirmed the microarray results. qPCR results of selected genes in the enriched Schwann cells were also coincident to microarray. Conversely, the regulation of *Foxo3* in the enriched fibroblasts of the sciatic nerve was in the opposite direction.

### Mitochondrial/oxidative stress mechanisms related to presymptomatic motor neuron impairment

Motor neurons are susceptible to reactive oxygen species and oxidative stress due to their high metabolic rates and decreased ability to buffer calcium (Shaw and Eggett, 2000). Long axons and increased functional activity also confer a high susceptibility to mitochondrial impairments in motor neurons (Barber et al., 2006; Pizzuti and Petrucci, 2011; Cozzolino et al., 2013).

Altered morphology of motor neuron mitochondria has been described in an ALS mouse model (Parone et al., 2013) as well as in humans (Hirano et al., 1984b) at both histological (Hirano et al., 1984a,b) and ultrastructural (Sasaki and Iwata, 1996) levels. Of note, swollen and vacuolated mitochondria were found in motor neurons, glial cells and muscles in ALS (Afifi et al., 1966; Atsumi, 1981; Siklós et al., 1996; Sasaki et al., 2007; Cassina et al., 2008).

Furthermore, oxidative stress has been described in several regions of the brain (Ferrante et al., 1997; Bogdanov et al., 1998, 2000), in the spinal cord (Shaw et al., 1995; Andrus et al., 1998; Liu et al., 1998, 2004; Shibata et al., 2002) and in the skeletal muscle (Mahoney et al., 2006) of both ALS mice and humans. Altered levels of reactive oxygen species within the spinal cord mitochondria of ALS mice (Jung et al., 2002) and patients (Bogdanov et al., 2000) support a possible impairment of the electron transport chain and energy defects in this disorder. Indeed, mitochondrion-induced damage to motor neurons has previously been mentioned as an early ALS signal (Wong et al., 1995; Mattiazzi et al., 2002; Kirkinezos et al., 2005; Cassina et al., 2008; Loizzo et al., 2010). Furthermore, peroxynitrite and superoxide overload in reactive astrocytes and microglia in *in vitro* models of ALS lead to protein impairment-induced motor neuron damage (Hensley et al., 2006; Dadon-Nachum et al., 2011). These studies all underline the critical involvement of mitochondrial dysfunction in early autonomous and non-autonomous pathogenesis of ALS.

Interestingly, increases in inducible nitric oxide synthase and peroxynitrite in Schwann cells and motor axons of paranodal regions in presymptomatic ALS mice were associated with local mitochondrial reactive oxygen species formation (Chen et al., 2010). Furthermore, Schwann cell-induced trophic support failure and Schwann cell-induced mitochondrial toxicity to motor neurons have both been correlated with high levels of mSOD1 in those peripheral glia of presymptomatic SOD1^G93A^ ALS mice (Gould et al., 2006). Thus, Schwann cell mitochondrion/oxidative stress mechanisms seem to play a key role during the early stages of ALS.

### KEGG pathways related to death, stress and mitochondrion in ALS

Our DAVID analyses identified genes associated with death, stress and mitochondrial function from the complete list of dysregulated genes of the sciatic nerve from 60-day-old presymptomatic ALS mice before the onset of neurological impairment.

Dysregulated genes of death signaling (Olsen et al., 2001; Hensley et al., 2002; Yoshihara et al., 2002; Dangond et al., 2004; Ferraiuolo et al., 2007; Guipponi et al., 2010) and also those of stress and mitochondrion-related signaling (Dangond et al., 2004; D'arrigo et al., 2010; Guipponi et al., 2010; Bernardini et al., 2013) have been described in ALS models (spinal cord and motor neurons) and in patients (e.g., from *post mortem* spinal cord and skeletal muscle biopsy) by means of microarray technology. Thus, our work supplements those cited by providing a large-scale profile of genes related to death, stress and mitochondrial function in the peripheral motor nerves of presymptomatic ALS mice.

KEGG pathways from the dysregulated genes associated with death, stress and mitochondrial function of sciatic nerves of presymptomatic ALS mice identified valine, leucine and isoleucine degradation and propionate metabolism as pathways with the highest number of dysregulated genes. Of note, impairments in valine, leucine and isoleucine metabolism (Ilzecka et al., 2003) but not in propionate metabolism, have been described in the context of ALS. Furthermore, fatty acid metabolism, citrate cycle, gluthatione metabolism, arginine and proline metabolism, pyruvate metabolism, sphingolipid metabolism, synthesis and degradation of ketone bodies, and beta-alanine metabolism pathways have been correlated with the onset of ALS (Ariga et al., 1998; Schulz et al., 2000; Cutler et al., 2002; Zhao et al., 2006; Nunn and Ponnusamy, 2009; Panov et al., 2011; Dormann et al., 2012; Zhang and Chook, 2012; de Munck et al., 2013; Yip et al., 2013; Allen et al., 2014). Additionally, dysregulated genes related to RIG-I-like receptor signaling (Day et al., 2005; Katsuno et al., 2011; Zhu et al., 2014), tryptophan metabolism (Sandyk, 2006), ErbB signaling (Gorlewicz et al., 2009), and cell cycle (Manzano et al., 2013) have been reported in ALS (Gorlewicz et al., 2009; Manzano et al., 2013).

Interestingly, the majority of dysregulated genes of the KEGG pathways RIG-I-like receptor signaling, ErbB signaling and cell cycle were found to be up-regulated. Conversely, all genes of tryptophan metabolism were down regulated in the sciatic nerve of presymptomatic ALS mice. The understanding on how such a complex regulation of important singling pathways may represent early triggering or reactive responses in the dying-back mechanisms in ALS is a matter for further analyses. RIG-I-like receptor genes are regulated in ALS, however, the exact mechanisms by which innate immune activation may drive neuronal death in neurodegenerative disorders are far from elucidated. In particular, the role of RIG-I-like receptor gene products in those mechanisms underlying glial cell activation, misfolded proteins/aberrantly localized nucleic acids and mitochondrion signaling-induced autophagy remain unclear (Kawai and Akira, 2008; Tal and Iwasaki, 2009; Heneka et al., 2014; Ying et al., 2015). Furthermore, the involvement of ErbB signaling in ALS was raised with the description of disrupted neuregulin-ErbB4 pathway signaling in presynaptic synapses of less resistant spinal cord motor neurons (Takahashi et al., 2013), but not in the resistant oculomotor neurons in human ALS (Gallart-Palau et al., 2014). Furthermore, ErbB signaling may influence non-autonomous microglial ALS mechanisms (Falls, 2003; Esper et al., 2006; Song et al., 2012) as well as Schwann cell-induced motor axon terminal changes in ALS (Gorlewicz et al., 2009).

The large-scale down-regulation of genes associated with the tryptophan metabolism pathway may be related to serotonin's ability to modulate glutamate motor neuron transmission (Palchaudhuri and Flügge, 2005), and thus excitotoxicity in ALS. Indeed, evidence of serotonin deregulation has been obtained from studies on ALS patients (Sandyk, 2006).

As discussed below, cell cycle gene regulation in the sciatic nerve in presymptomatic phases of ALS might disrupt the normal interactions between reactive Schwann cells and motor axons. In fact, cell cycle impairments have been described in neurodegenerative diseases (Van Leeuwen and Hoozemans, 2015; Wojsiat et al., 2015) and the interaction between mSOD1 and cyclin regulators seems to contribute to autonomous and non-autonomous mechanisms in ALS (Nguyen et al., 2003; Ranganathan and Bowser, 2003; Cova et al., 2010).

### Schwann cell genes related to death, stress and mitochondrial function in the sciatic nerve of presymptomatic ALS mouse model

Our study demonstrated the existence of highly interconnected genes from the lists of dysregulated genes involved in death and stress signaling as well as mitochondrial function in the sciatic nerve of presymptomatic ALS mice. The regulation of these genes was further evaluated in the enriched Schwann cells from the ALS mice using qPCR.

*Traf2, E2f1*, and *Cdkn1b* from the list of genes of GO biological process related to death were identified as nodes with highest values for node degree (number of connections) and node betweenness (number of shortest paths) in the Protein interaction network analysis.

Mutant SOD1 led to TNF-α pathway activation in the absence of inflammation in ALS (Carter et al., 2009), an event that may also occur in Schwann cells (Au and Yeh, 2007) based on the results of *Traf2* up-regulation in ALS Schwann cells described in our work.

TNFα is a potent regulator of Schwann cell division (Chandross et al., 1996) and activation (Bonetti et al., 2000). Moreover, despite a lack of histopathological descriptions of peripheral nerves from ALS subjects and animal models (Fischer et al., 2004; Kano et al., 2012), there are no reports describing local Schwann cell division or death. However, up-regulation of markers of glial activation indicated an early process of Schwann cell reaction in ALS peripheral nerves (Keller et al., 2009; Malaspina et al., 2010). Cell division is a common feature of activated central glia (Pekny and Nilsson, 2005; Dheen et al., 2007). Therefore, it might be possible that the absence of cell division would disrupt normal functions of activated Schwann cells in presymptomatic stages of ALS.

It is likely that the up-regulation *Cdkn1b* and *E2f-1* may lead to intracellular mobilization of cell-cycle proteins and transcriptional regulators in ALS glia (Weinberg, 1995; Ranganathan and Bowser, 2003) and also in ALS activated Schwann cells. These events may interfere with the balance between death/survival signaling pathways in TNFα-activated Schwann cells (Tang et al., 2013), possibly altering the normal functions of activated Schwann cells and leading to a failure of trophic surveillance and/or to toxicity signaling in ALS (Ranganathan and Bowser, 2003; Cova et al., 2010). Therefore, the absence of hyperplasia may impair substantially the paracrine trophic mechanisms of TNFα-activated Schwann cells with motor neurons in ALS.

Other nodes with high values for connections and betweenness described in the Protein interaction network analysis support a non-autonomous mechanism of activated Schwann cells in ALS. The nodes *Ngfr, Erbb3*, and *H2afx* of dysregulated genes from the death list further indicated the presence of impaired paracrine trophic functions of Schwann cells in ALS. The up-regulation of *Ngfr* and *Erbb3*, which encode the high-affinity neurotrophin receptor TRKA and the neuregulin-associated tyrosine kinase receptor, respectively, could be involved in Schwann cell paracrine functions (Wang et al., 1996; Lyons et al., 2005; Adilakshmi et al., 2011) as well as in early, complex mechanisms of axonal retraction and neuromuscular junction alterations in ALS prior to motor neuron degeneration (Kerkhoff et al., 1991; Gorlewicz et al., 2009). Furthermore, the up-regulation of *H2afx* in Schwann cells might correlate with the described Schwann cell participation in the pathogenesis of ALS since H2AFX-induced DNA damage in reactive astrocytes has been associated with impaired paracrine glial functions in other neurodegenerative disorder (Simpson et al., 2010).

*Foxo3* and *Cdkn1a* are present in the death and stress lists of genes and were also found to be dysregulated in the enriched Schwann cells from the sciatic nerve of 60-day-old presymptomatic ALS mice. Indeed, the encoded products of these genes have been investigated in ALS skeletal muscles but not in the ALS motor nerve (Léger et al., 2006; Gonzalez de Aguilar et al., 2008; Manzano et al., 2013). *Foxo3* showed high levels of betweenness and degree and FOXO3 has been studied as a catabolic target in ALS skeletal muscles (Léger et al., 2006). Conversely, *Cdkn1a* dysregulation was described in muscles of ALS mice (Gonzalez de Aguilar et al., 2008) and CDKN1A, a cyclin-dependent kinase inhibitor, interferes with satellite cell-induced myoplasticity in ALS skeletal muscles (Manzano et al., 2013). The above findings provide support for the involvement of *Foxo3* and *Cdkn1a* in the dying-back Schwann cell mechanisms in ALS, a matter that should be further investigated.

*Hspa1a, Prdx2*, and *Rrm2b* were present in three GO lists, the death, stress, and mitochondrial function categories. *Hspa1a* and *Prdx2* were also found to be dysregulated in the enriched Schwann cells from the sciatic nerves of presymptomatic ALS mice. Those three genes and their encoded products have not been described in the context of ALS. Moreover, up-regulation of *Hspa1a* and *Prdx2* were found in other neurodegenerative disorders and their encoded products have been studied in the context of therapeutic strategies (Muchowski and Wacker, 2005; Ali et al., 2010; Gestwicki and Garza, 2012). Furthermore, RRM2B-related mitochondrial diseases are frequently inherited and associated with neurological symptoms (Horga et al., 2014). Conversely, non-inherited mitochondrial dysfunctions have been suggested as possible mechanisms underlying the pathogenesis of ALS (discussed above). Thus, mitochondria/oxidative stress impairing Schwann cell paracrine regulation in the presymptomatic phases of ALS is a matter for further investigation.

*Mtor*, identified in the mitochondrion list of dysregulated genes, was down-regulated in the enriched Schwann cells from presymptomatic SOD1 mice, a change which is in line with descriptions of MTOR reduction in spinal cords of ALS rodents (Morimoto et al., 2007). MTOR reduction induced by PI3-K and Akt/PKB signaling (Nagano et al., 2002) worsened ALS pathology in ALS transgenic mice (Zhang et al., 2011). Because MTOR activation leads to neuroprotection in ALS (Saxena et al., 2013), it is a matter of further investigation whether modulation of MTOR signaling in Schwann cells could counteract the degenerative processes associated with this disease.

Finally, *Mapk10* of mitochondrion and stress categories was found to be up-regulated in the enriched Schwann cells from sciatic nerves of presymptomatic ALS mice. A MAPK10/JNK3 truncation mutation has previously only been associated with cognitive disorders (Kunde et al., 2013). Furthermore, *Mapk10* itself has not been investigated as a factor in the pathogenesis of ALS, despite the general consensus that deregulated MAPK signaling contributes to ALS (Kim and Choi, 2010; Xia et al., 2015). Nevertheless, the involvement of p38MAPK and TNFα receptors in the non-autonomous microglial toxicity in ALS (Veglianese et al., 2006) raised the possibility of a similar mechanism in ALS Schwann cells, an issue that should be explored further.

In conclusion, our large-scale gene profiling reveled the presence of death, stress (with emphasis on oxidative stress), and mitochondrial function pathway signaling taking place in the sciatic nerve of presymptomatic ALS mice. The regulation of highly interconnected genes in ALS Schwann cells indicated the involvement of these pathways in the non-autonomous mechanisms of that peripheral glial cell type in the early stages of the disorder.

## Author contributions

JM and GC designed the study. CA and JM performed the experiments. GC interpreted the results. All authors wrote, read and approved the final manuscript. GC is responsible for the ALS Brazil Project of the University of São Paulo School of Medicine.

### Conflict of interest statement

The authors declare that the research was conducted in the absence of any commercial or financial relationships that could be construed as a potential conflict of interest.
