# Supplementary material for: Dysregulated expression of death, stress and mitochondrion related genes in the sciatic nerve of presymptomatic SOD1G93A mouse model of Amyotrophic Lateral Sclerosis
Source: Front Cell Neurosci. 2015 Sep 1;9:332. doi: 10.3389/fncel.2015.00332 (PMC4555015; doi:10.3389/fncel.2015.00332)
Supplement: Supplementary file 1 [file DataSheet1.PDF]

Original Research

Dysregulated expression of death, stress and mitochondrion related genes in the sciatic nerve of presymptomatic SOD1^{G93A} mouse model of Amyotrophic Lateral Sclerosis

Chrystian Junqueira Alves, Jessica Ruivo Maximino and Gerson Chadi*

(Alves CJ, Maximino JR, Chadi G)

Neuroregeneration Center, Department of Neurology, University of São Paulo School of Medicine, São Paulo, Brazil.

*Corresponding author:
Gerson Chadi. M.D., Ph.D.
Full Professor
Department of Neurology
University of São Paulo
Av. Dr. Arnaldo, 455, 2nd floor, room 2119
01246-903-São Paulo. Brazil
Phone: 55 11 3061-7460
E-mail address: gerchadi@usp.br

Quantitative PCR in Fibroblasts

Total RNA from enriched fibroblast was extracted using Trizol and synthesized in cDNA as described in the text. qPCR reactions to *Foxo3* were carried out in duplicate with 10 ng cDNA, using the DyNAmo ColorFlash SYBR Green qPCR kit (Thermo Scientific, USA) and 400 nM of each primer in a final reaction volume of 20 µl. Reactions were run with the Applied Biosystems 7500 Real-Time PCR System (Applied Biosystems) as described in the text. Sequence information regarding the SYBR primers can be found in Table 1 in the manuscript. Gene expression was normalized to the expression of *Actb* and determined using the ΔΔCt mathematical model as described in the text.

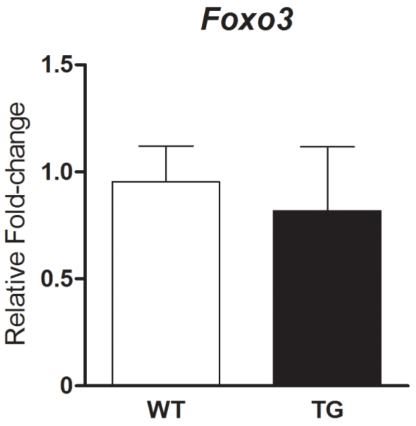

Figure S1. Relative fold change values of *Foxo3* by qPCR in enriched fibroblast samples of 60-day-old SOD1^{G93A} mice compared to the age matched wild-type (WT) controls. Fibroblasts were enriched by means of flow cytometry cell sorting technique from the sciatic nerve of the mice. No difference in the expression of the gene is seen. *Foxo3* is a selected gene of the Death and Stress categories. Means ± SEM, according to unpaired two-tailed t-test. *n*=6 of each group.

Table S1. List of differentially expressed genes in sciatic nerve of 60 days old SOD1^{G93A} mice related to death, stress and mitochondrion obtained from enriched analysis.

Probe Set ID	Gene Symbol	Gene name
A_55_P2015495	*Abat*	4-aminobutyrate aminotransferase
A_51_P245368	*Abcb1b*	ATP-binding cassette, sub-family B (MDR/TAP), member 1B
A_55_P2176489	*Abcd1*	ATP-binding cassette, sub-family D (ALD), member 1
A_51_P125260	*Acaa2*	acetyl-Coenzyme A acyltransferase 2
A_55_P2084703	*Acaca*	acetyl-Coenzyme A carboxylase alpha
A_55_P2019054	*Acacb*	acetyl-Coenzyme A carboxylase beta
A_55_P2013858	*Acad8*	acyl-Coenzyme A dehydrogenase family, member 8
A_51_P435068	*Acadsb*	acyl-Coenzyme A dehydrogenase, short/branched chain
A_51_P319449	*Acat1*	acetyl-Coenzyme A acetyltransferase 1
A_66_P101407	*Acat3*	acetyl-Coenzyme A acetyltransferase 3
A_52_P367675	*Acin1*	apoptotic chromatin condensation inducer 1
A_55_P2082914	*Acly*	ATP citrate lyase
A_55_P2085880	*Aco1*	aconitase 1
A_51_P487175	*Acsm3*	acyl-CoA synthetase medium-chain family member 3
A_52_P57622	*Acss3*	acyl-CoA synthetase short-chain family member 3
A_52_P629895	*Adh1*	alcohol dehydrogenase 1 (class I)
A_52_P116264	*Adhfe1*	alcohol dehydrogenase, iron containing, 1
A_55_P2145804	*Aen*	apoptosis enhancing nuclease
A_55_P2145804	*Aen*	apoptosis enhancing nuclease
A_51_P227770	*Agxt2l2**	alanine-glyoxylate aminotransferase 2-like 2
A_55_P2002859	*Aifm2*	apoptosis-inducing factor, mitochondrion-associated 2
A_55_P2002859	*Aifm2*	apoptosis-inducing factor, mitochondrion-associated 2
A_52_P517289	*Akap1*	A kinase (PRKA) anchor protein 1
A_55_P2083649	*Alas1*	aminolevulinic acid synthase 1
A_51_P215887	*Aldh18a1*	aldehyde dehydrogenase 18 family, member A1
A_51_P338485	*Aldh6a1*	aldehyde dehydrogenase family 6, subfamily A1
A_52_P265877	*Aldh9a1*	aldehyde dehydrogenase 9, subfamily A1
A_55_P2016682	*Alkbh7*	alkB, alkylation repair homolog 7

A_55_P2454521	*Alms1*	Alstrom syndrome 1 homolog (human)
A_51_P520306	*Alox12*	arachidonate 12-lipoxygenase
A_51_P149562	*Apbb2*	amyloid beta (A4) precursor protein-binding, family B, member 2
A_55_P2049582	*Apbb3*	amyloid beta (A4) precursor protein-binding, family B, member 3
A_55_P1998506	*Aplf*	aprataxin and PNKP like factor
A_51_P171999	*Apoe*	apolipoprotein E
A_55_P2048409	*Apool*	apolipoprotein O-like
A_55_P2011137	*Armc10*	armadillo repeat containing 10
A_52_P436643	*Asah2*	N-acylsphingosine amidohydrolase 2
A_52_P436643	*Asah2*	N-acylsphingosine amidohydrolase 2
A_51_P437327	*Ascl1*	achaete-scute complex homolog 1 (Drosophila)
A_55_P2143070	*Ass1*	argininosuccinate synthetase 1
A_51_P371174	*Bag3*	BCL2-associated athanogene 3
A_55_P1999419	*Bag5*	BCL2-associated athanogene 5
A_55_P2186648	*Bard1*	BRCA1 associated RING domain 1
A_51_P446570	*Bbox1*	butyrobetaine (gamma), 2-oxoglutarate dioxygenase 1
A_51_P400659	*Bcap29*	B-cell receptor-associated protein 29
A_55_P1960167	*Bcat2*	branched chain aminotransferase 2, mitochondrial
A_51_P466148	*Bckdha*	branched chain ketoacid dehydrogenase E1, alpha polypeptide
A_55_P2066116	*Bcl3*	B-cell leukemia/lymphoma 3
A_52_P161495	*Bcl6*	B-cell leukemia/lymphoma 6
A_55_P2053978	*Bdkrb2*	bradykinin receptor, beta 2
A_55_P2154387	*Bmp4*	bone morphogenetic protein 4
A_55_P2091473	*Bnip3***	BCL2/adenovirus E1B interacting protein 3
A_55_P1969341	*Brip1*	BRCA1 interacting protein C-terminal helicase 1
A_55_P2051991	*Brsk1*	BR serine/threonine kinase 1
A_52_P31543	*Btg2*	B-cell translocation gene 2, anti-proliferative
A_51_P191611	*Cat**	catalase
A_55_P1962419	*Ccar1*	cell division cycle and apoptosis regulator 1
A_52_P244193	*Cd24a*	CD24a antigen
A_55_P2121352	*Cdk5*	cyclin-dependent kinase 5
A_51_P363947	*Cdkn1a*	cyclin-dependent kinase inhibitor 1A (P21)
A_52_P673863	*Cdkn1b*	cyclin-dependent kinase inhibitor 1B
A_52_P159050	*Cds2*	CDP-diacylglycerol synthase (phosphatidate cytidylyltransferase) 2
A_55_P2386587	*Cep63*	centrosomal protein 63
A_55_P2146177	*Cerk**	ceramide kinase
A_51_P446978	*Cfdp1*	craniofacial development protein 1
A_51_P254234	*Chchd4*	coiled-coil-helix-coiled-coil-helix domain containing 4
A_55_P1963198	*Chchd7*	coiled-coil-helix-coiled-coil-helix domain containing 7
A_55_P2030732	*Cln3*	ceroid lipofuscinosis, neuronal 3, juvenile
A_55_P1959036	*Cox16*	COX16 cytochrome c oxidase assembly homolog (S. cerevisiae)
A_51_P480499	*Cox4nb*	COX4 neighbor
A_51_P257258	*Cpox*	coproporphyrinogen oxidase
A_55_P2057405	*Cpt1a**	carnitine palmitoyltransferase 1a, liver
A_55_P2033660	*Crls1*	cardiolipin synthase 1
A_55_P2016540	*Cry2*	cryptochrome 2 (photolyase-like)
A_52_P672803	*Ctsa*	cathepsin A
A_51_P465148	*Ctsb*	cathepsin B
A_51_P384629	*Ctsd*	cathepsin D
A_51_P196925	*Cx3cl1*	chemokine (C-X3-C motif) ligand 1
A_55_P2007964	*Cx3cr1*	chemokine (C-X3-C) receptor 1
A_55_P2090214	*Cyb5r2*	cytochrome b5 reductase 2
A_55_P2065866	*Cygb*	cytoglobin
A_51_P308347	*Dact2*	dapper homolog 2, antagonist of beta-catenin (xenopus)
A_51_P391955	*Dapl1*	death associated protein-like 1
A_55_P2021744	*Dcc*	deleted in colorectal carcinoma
A_55_P2159189	*Decr1**	2,4-dienoyl CoA reductase 1, mitochondrial
A_55_P2066414	*Dffa*	DNA fragmentation factor, alpha subunit
A_55_P1958230	*Dhodh*	dihydroorotate dehydrogenase
A_55_P1966159	*Dlg5*	discs, large homolog 5 (Drosophila)
A_55_P1985835	*Dnase1*	deoxyribonuclease I
A_52_P444457	*Dnlz*	DNL-type zinc finger
A_52_P164286	*Dnm3*	dynamin 3
A_52_P89064	*Dusp18*	dual specificity phosphatase 18
A_55_P2317665	*E2f1*	E2F transcription factor 1
A_55_P2142439	*Echdc2**	enoyl Coenzyme A hydratase domain containing 2

A_51_P462918	*Ehhadh*	dehydrogenase
A_52_P559919	*Eif2ak2*	eukaryotic translation initiation factor 2-alpha kinase 2
A_55_P1998396	*Eif2ak4*	eukaryotic translation initiation factor 2 alpha kinase 4
A_51_P250058	*Epas1*	endothelial PAS domain protein 1
A_55_P2119897	*Erbb3*	v-erb-b2 erythroblastic leukemia viral oncogene homolog 3 (avian)
A_55_P2060747	*Esco1*	establishment of cohesion 1 homolog 1 (S. cerevisiae)
A_55_P2029678	*Etfa*	electron transferring flavoprotein, alpha polypeptide
A_55_P2105200	*Fam175a*	family with sequence similarity 175, member A
A_55_P2058831	*Fancc*	Fanconi anemia, complementation group C
A_51_P142346	*Fancm*	Fanconi anemia, complementation group M
A_55_P2008740	*Fcgr1*	Fc receptor, IgG, high affinity I
A_52_P623751	*Fem1b*	feminization 1 homolog b (C. elegans)
A_55_P2035122	*Fitm2*	fat storage-inducing transmembrane protein 2
A_51_P196444	*Foxc2*	forkhead box C2
A_55_P2041668	*Foxl2*	forkhead box L2
A_55_P2129309	*Foxo3*	forkhead box O3
A_51_P296608	*Gadd45a*	growth arrest and DNA-damage-inducible 45 alpha
A_55_P2150633	*Gadd45gip1*	growth arrest and DNA-damage-inducible
A_51_P129149	*Gatad2a**	GATA zinc finger domain containing 2A
A_51_P228276	*Gbas*	glioblastoma amplified sequence
A_51_P159402	*Gcat*	glycine C-acetyltransferase
A_51_P365019	*Gclc*	glutamate-cysteine ligase, catalytic subunit
A_51_P365019	*Gclc*	glutamate-cysteine ligase, catalytic subunit
A_66_P127070	*Gdf5*	growth differentiation factor 5
A_55_P2045896	*Gdnf*	glial cell line derived neurotrophic factor
A_52_P482251	*Gjb6*	gap junction protein, beta 6
A_51_P171288	*Gli3*	GLI-Kruppel family member GLI3
A_55_P2108171	*Glud1*	glutamate dehydrogenase 1
A_55_P2146577	*Gm13570*	predicted gene 13570
A_55_P2067645	*Gm5136*	predicted gene 5136
A_55_P1968370	*Gprc5c*	G protein-coupled receptor, family C, group 5, member C
A_55_P1983152	*Gramd4**	GRAM domain containing 4
A_55_P1988708	*Gstz1*	glutathione transferase zeta 1 (maleylacetoacetate isomerase)
A_66_P108622	*Gtpbp10*	GTP-binding protein 10 (putative)
A_55_P2135526	*Gzmc*	granzyme C
A_55_P2150757	*Gzmm*	granzyme M (lymphocyte met-ase 1)
A_51_P245275	*H2afx*	H2A histone family, member X
A_55_P2106429	*Herc2*	hect (homologous to the E6-AP (UBE3A)
A_55_P2087013	*Hipk1*	homeodomain interacting protein kinase 1
A_55_P2091858	*Hipk2*	homeodomain interacting protein kinase 2
A_55_P1992582	*Hmgcs2**	3-hydroxy-3-methylglutaryl-Coenzyme A synthase 2
A_55_P2171158	*Hmgn1*	high mobility group nucleosomal binding domain 1
A_55_P2068459	*Hspa1a*	heat shock protein 1A
A_55_P2076772	*Hspa5*	heat shock protein 5
A_52_P515036	*Htatip2*	HIV-1 tat interactive protein 2, homolog (human)
A_55_P1973698	*Htra2*	HtrA serine peptidase 2
A_55_P2088530	*Htt*	huntingtin
A_51_P108935	*Iars2*	isoleucine-tRNA synthetase 2, mitochondrial
A_51_P132978	*Idh1*	isocitrate dehydrogenase 1 (NADP+), soluble
A_51_P268559	*Idh3a*	isocitrate dehydrogenase 3 (NAD+) alpha
A_52_P52156	*Isoc2a*	isochorismatase domain containing 2a
A_51_P343356	*Isoc2b*	isochorismatase domain containing 2b
A_55_P2097913	*Itga10*	integrin, alpha 10
A_52_P216672	*Klk8*	kallikrein related-peptidase 8
A_55_P2128501	*Krt8*	keratin 8
A_55_P2170009	*Lactb2*	lactamase, beta 2
A_55_P1984035	*Litaf**	LPS-induced TN factor
A_55_P2052834	*Lst1*	leukocyte specific transcript 1
A_51_P302566	*Maob*	monoamine oxidase B
A_55_P2070529	*Mapk10*	mitogen-activated protein kinase 10
A_52_P252258	*Mapk12*	mitogen-activated protein kinase 12
A_51_P396385	*Mars2*	methionine-tRNA synthetase 2 (mitochondrial)
A_52_P599264	*Mdfic*	MyoD family inhibitor domain containing
A_51_P394676	*Mef2d*	myocyte enhancer factor 2D
A_55_P2175880	*Mgst1*	microsomal glutathione S-transferase 1
A_51_P246727	*Mlxip*	MLX interacting protein

A_51_P270364	*Mmaa*	methylmalonic aciduria (cobalamin deficiency) type A
A_55_P2129319	*Mre11a*	meiotic recombination 11 homolog A (S. cerevisiae)
A_66_P136372	*Mrpl9*	mitochondrial ribosomal protein L9
A_55_P1970740	*Mrps10*	mitochondrial ribosomal protein S10
A_51_P198292	*Mrps2*	mitochondrial ribosomal protein S2
A_55_P2101426	*Mrps25*	mitochondrial ribosomal protein S25
A_51_P283708	*Msh2*	mutS homolog 2 (E. coli)
A_55_P2064507	*Msra*	methionine sulfoxide reductase A
A_51_P431870	*Mtap1s*	microtubule-associated protein 1S
A_52_P601958	*Mtch2*	mitochondrial carrier homolog 2 (C. elegans)
A_52_P184149	*Mthfd2*	methylenetetrahydrofolate dehydrogenase (NAD+ dependent)
A_52_P67643	*Mtor*	mechanistic target of rapamycin (serine/threonine kinase)
A_65_P05358	*Nd1*	NADH dehydrogenase subunit 1
A_55_P2039110	*Nd2*	NADH dehydrogenase subunit 2
A_51_P245525	*Nd4*	NADH dehydrogenase subunit 4
A_55_P1990648	*Ndufaf4*	NADH dehydrogenase (ubiquinone) 1 alpha subcomplex, assembly factor 4
A_52_P251366	*Neil3*	nei like 3 (E. coli)
A_51_P315646	*Nfs1*	nitrogen fixation gene 1 (S. cerevisiae)
A_52_P236448	*Ngfr*	nerve growth factor receptor (TNFR superfamily, member 16)
A_52_P128964	*Nhej1*	nonhomologous end-joining factor 1
A_55_P1958349	*Nipsnap3b*	nipsnap homolog 3B (C. elegans)
A_51_P172532	*Nit1*	nitrilase 1
A_52_P66371	*Nlrx1*	NLR family member X1
A_51_P164203	*Nme4*	non-metastatic cells 4
A_55_P2158866	*Nme6*	non-metastatic cells 6
A_55_P2129608	*Nrp*	neural regeneration protein
A_51_P507023	*Nt5dc3*	5'-nucleotidase domain containing 3
A_52_P510107	*Nudt15*	nudix (nucleoside diphosphate linked moiety X)-type motif 15
A_51_P111952	*Nudt19*	nudix (nucleoside diphosphate linked moiety X)-type motif 19
A_66_P123683	*Obfc2a*	oligonucleotide/oligosaccharide-binding fold containing 2A
A_51_P228193	*Ociad1*	OCIA domain containing 1
A_55_P2128511	*Ociad2*	OCIA domain containing 2
A_51_P256945	*Opa3*	optic atrophy 3 (human)
A_52_P121	*Osgepl1*	O-sialoglycoprotein endopeptidase-like 1
A_51_P291361	*Osm*	oncostatin M
A_55_P2134012	*Oxa1l*	oxidase assembly 1-like
A_66_P108770	*Oxct1*	3-oxoacid CoA transferase 1
A_55_P2077133	*Oxsr1*	oxidative-stress responsive 1
A_55_P2117704	*Pak7*	p21 protein (Cdc42/Rac)-activated kinase 7
A_52_P247943	*Parl**	presenilin associated, rhomboid-like
A_55_P2148518	*Pax2*	paired box gene 2
A_55_P2113723	*Pecr*	peroxisomal trans-2-enoyl-CoA reductase
A_51_P195958	*Phlda1*	pleckstrin homology-like domain, family A, member 1
A_51_P329928	*Phlda3*	pleckstrin homology-like domain, family A, member 3
A_55_P1987439	*Pigt*	phosphatidylinositol glycan anchor biosynthesis, class T
A_55_P1954167	*Plagl2*	pleiomorphic adenoma gene-like 2
A_66_P129444	*Pnkp*	polynucleotide kinase 3'- phosphatase
A_55_P2023191	*Polg2*	polymerase (DNA directed), gamma 2, accessory subunit
A_55_P2367250	*Pou4f1*	POU domain, class 4, transcription factor 1
A_52_P116372	*Ppp1r15b*	protein phosphatase 1, regulatory (inhibitor) subunit 15b
A_55_P1979432	*Prdx2*	peroxiredoxin 2
A_51_P225793	*Prr5l**	proline rich 5 like
A_52_P605812	*Ptrh1*	peptidyl-tRNA hydrolase 1 homolog (S. cerevisiae)
A_55_P1987499	*Pttg1*	pituitary tumor-transforming gene 1
A_55_P2160712	*Pura**	purine rich element binding protein A
A_51_P287232	*Qars*	glutaminyl-tRNA synthetase
A_52_P392742	*Qtrtd1*	queuine tRNA-ribosyltransferase domain containing 1
A_55_P2094852	*Rabep1*	rabaptin, RAB GTPase binding effector protein 1
A_55_P1985749	*Rad23a*	RAD23a homolog (S. cerevisiae)
A_66_P120949	*Rad51l3*	RAD51-like 3 (S. cerevisiae)
A_51_P105017	*Rad52*	RAD52 homolog (S. cerevisiae)
A_52_P89567	*Rhob*	ras homolog gene family, member B
A_52_P232663	*Rhot1*	ras homolog gene family, member T1
A_52_P179785	*Ripk2*	receptor (TNFRSF)-interacting serine-threonine kinase 2
A_51_P491987	*Ripk3*	receptor-interacting serine-threonine kinase 3
A_51_P477019	*Rnaset2a*	ribonuclease T2A

A_52_P425706	*Rnf168*	ring finger protein 168
A_55_P2008815	*Rrm2b*	ribonucleotide reductase M2 B (TP53 inducible)
A_51_P514913	*Rsad1*	radical S-adenosyl methionine domain containing 1
A_55_P1994564	*Sap30bp*	SAP30 binding protein
A_55_P2128451	*Sfpq*	splicing factor proline/glutamine rich
A_51_P418526	*Sfxn1**	sideroflexin 1
A_55_P2066958	*Sh3glb1*	SH3-domain GRB2-like B1 (endophilin)
A_55_P2098354	*Shf*	Src homology 2 domain containing F
A_52_P49014	*Shh*	sonic hedgehog
A_51_P411909	*Sirt5*	sirtuin 5 (silent mating type information regulation 2 homolog) 5
A_66_P134481	*Skp2*	S-phase kinase-associated protein 2 (p45)
A_55_P1972187	*Slc25a14*	solute carrier family 25 (mitochondrial carrier, brain), member 14
A_51_P510437	*Slc25a15*	solute carrier family 25 (mitochondrial carrier ornithine transporter), member 15
A_51_P193173	*Slc25a25*	solute carrier family 25 (mitochondrial carrier, phosphate carrier) , member 25
A_51_P354652	*Slc25a30*	solute carrier family 25, member 30
A_51_P343429	*Slc25a37*	solute carrier family 25, member 37
A_55_P1964068	*Slc25a38*	solute carrier family 25, member 38
A_51_P383140	*Slk*	STE20-like kinase (yeast)
A_55_P2033958	*Sltm*	SAFB-like, transcription modulator
A_66_P115061	*Smc6*	structural maintenance of chromosomes 6
A_51_P501248	*Sphk1*	sphingosine kinase 1
A_55_P2131173	*Sphk2*	sphingosine kinase 2
A_55_P2033120	*Srxn1*	sulfiredoxin 1 homolog (S. cerevisiae)
A_52_P58181	*Suclg2*	succinate-Coenzyme A ligase, GDP-forming, beta subunit
A_51_P193716	*Supv3l1*	suppressor of var1, 3-like 1 (S. cerevisiae)
A_55_P2141301	*Synj2bp*	synaptojanin 2 binding protein
A_51_P508580	*Tcf15*	transcription factor 15
A_52_P351785	*Tfam*	transcription factor A, mitochondrial
A_65_P10913	*Tgfb2**	transforming growth factor, beta 2
A_55_P2137206	*Tgfbr1*	transforming growth factor, beta receptor I
A_51_P248265	*Thoc1*	THO complex 1
A_55_P2081616	*Timeless*	timeless homolog (Drosophila)
A_55_P1985850	*Timp1*	tissue inhibitor of metalloproteinase 1
A_51_P336721	*Tipin*	timeless interacting protein
A_51_P310398	*Tk2*	thymidine kinase 2, mitochondrial
A_55_P1979027	*Tmem65*	transmembrane protein 65
A_55_P2043932	*Tmem8b*	transmembrane protein 8B
A_51_P131408	*Tnfrsf12a*	tumor necrosis factor receptor superfamily, member 12a
A_55_P2018017	*Tnfsf10*	tumor necrosis factor (ligand) superfamily, member 10
A_51_P518156	*Tomm20l*	translocase of outer mitochondrial membrane 20 homolog (yeast)-like
A_55_P2032363	*Tomm40l*	translocase of outer mitochondrial membrane 40 homolog-like yeast)
A_51_P343833	*Traf1*	TNF receptor-associated factor 1
A_55_P2092963	*Traf2*	TNF receptor-associated factor 2
A_55_P2092968	*Traf3*	TNF receptor-associated factor 3
A_51_P106059	*Traf4*	TNF receptor associated factor 4
A_55_P2009988	*Trib3*	tribbles homolog 3 (Drosophila)
A_51_P468126	*Trim39*	tripartite motif-containing 39
A_52_P556082	*Trmt2b*	TRM2 tRNA methyltransferase 2 homolog B (S. cerevisiae)
A_55_P1973906	*Trp53inp1**	transformation related protein 53 inducible nuclear protein 1
A_52_P94454	*Trpc2*	transient receptor potential cation channel, subfamily C, member 2
A_55_P2078428	*Tstd1*	thiosulfate sulfurtransferase (rhodanese)-like domain containing 1
A_52_P20906	*Twist1*	twist homolog 1 (Drosophila)
A_51_P438805	*Txnip*	thioredoxin interacting protein
A_55_P2112111	*Ube2b*	ubiquitin-conjugating enzyme E2B, RAD6 homology (S. cerevisiae)
A_52_P434974	*Ubqln1*	ubiquilin 1
A_55_P2178800	*Ugt1a10*	UDP glycosyltransferase 1 family, polypeptide A10
A_55_P2035286	*Uhrf1*	ubiquitin-like, containing PHD and RING finger domains, 1
A_51_P424532	*Vnn1*	vanin 1
A_55_P1967867	*Xpa**	xeroderma pigmentosum, complementation group A
A_51_P392705	*Xpc*	xeroderma pigmentosum, complementation group C
A_55_P2016316	*Xrn2*	5'-3' exoribonuclease 2
A_51_P312997	*Zfp346*	zinc finger protein 346

Enriched analysis from microarray experiment of sciatic nerve of 60-day-old SOD1^{G93A} mice and their wild-type controls pointed differentially expressed genes related to death, stress and mitochondrion. * two and ** three probes for the same gene.

Table S2. Differentially expressed genes related to death, stress and mitochondrion with fold between -1.25 and 1.25 in sciatic nerve of 60 days-old SOD1^{G93A} mice.

Genes Related to Death

Gene	Fold	Ref
Krt8	-1.25	
Mef2d	-1.24	
Qars	-1.22	
Bcap29	-1.19	
Apbb2	-1.17	
Phlda3	-1.16	
Ube2b	-1.15	
Cfdp1	1.13	
Sh3glb1	1.14	
Dlg5	1.16	
Cdk5	1.16	[1,2,3,4,5]
Litaf	1.16	
Bag3	1.17	[6,7,8]
Sltm	1.18	
Tgfbr1	1.20	
Aen	1.20	
Traf3	1.20	
Ubqln1	1.21	
Msh2	1.22	
Sap30bp	1.23	
Htt	1.23	
Trim39	1.25	

GO	Term
GO:0010941	regulation of cell death
GO:0043067	regulation of programmed cell death
GO:0042981	regulation of apoptosis
GO:0016265	death
GO:0008219	cell death
GO:0012501	programmed cell death
GO:0006915	apoptosis
GO:0043069	negative regulation of programmed cell death
GO:0060548	negative regulation of cell death
GO:0043066	negative regulation of apoptosis
GO:0010942	positive regulation of cell death
GO:0043068	positive regulation of programmed cell death

Genes Related to Stress

Gene	Fold	Ref
Idh1	-1.25	
Phlda3	-1.16	
Smc6	-1.16	
Ube2b	-1.15	
Srxn1	1.19	
Brsk1	1.20	
Aen	1.20	
Rnf168	1.21	
Msh2	1.22	
Gm5136	1.23	
Hmgn1	1.24	

GO	Term
GO:0033554	cellular response to stress
GO:0006979	response to oxidative stress

Genes Related to Mitochondrion

Gene	Fold	Ref
Idh1	-1.25	
Acaa2	-1.24	
Bckdha	-1.24	
Abcb1b	-1.23	
Nit1	-1.23	
Acat1	-1.21	
Slc25a37	-1.21	
Glud1	-1.19	
Aldh9a1	-1.19	
Iars2	-1.19	
Acadsb	-1.19	
Idh3a	-1.19	
Nd4	-1.19	
Dhodh	-1.17	
Oxa1l	-1.16	
Gbas	-1.16	
Tk2	-1.15	
Sh3glb1	1.14	
Apool	1.15	
Dnlz	1.17	
Oxct1	1.18	
Nt5dc3	1.19	
Tmem8b	1.19	
Abat	1.19	
Crls1	1.19	
Armc10	1.20	
Gcat	1.21	
Mrps25	1.22	
Ptrh1	1.24	
Mmaa	1.24	
Slc25a14	1.25	
Trim39	1.25	
Ndufaf4	1.25	
Ociad2	1.25	

GO	Term
GO:0005739	mitochondrion
GO:0005741	mitochondrial outer membrane

Enriched analysis by means DAVID tool pointed genes from Gene Ontology (GO) Biological Processes terms related to Death and Stress, and Cell Components related to Mitochondrion. Positive and negative values pointed to down and upregulated genes expressions, respectively. References report to previous publications of respective genes or their related gene products in the context of Amyotrophic Lateral Sclerosis: 1) Bajaj, 2000; 2) Dobrowolny et al., 2008; 3) Kriz et al., 2003; 4) Nguyen et al., 2001; 5) Park and Vincent, 2008; 6) Crippa et al., 2010; 7) Crippa et al., 2013a; 8) Crippa et al., 2013b.

Protein interaction network analysis

Using the Cytoscape plugin GeneMania, we generated separately networks showing the interactions between those genes related to death (Figure S2), stress (Figure S3) and mitochondrion (Figure S4). The highly connected (hubs; degree values) and central (bottlenecks; betweenness values) related to death and stress are shown in a scatter plot (Figure S5). The network based on the differentially expressed genes related to death identified 2 highly connected nodes (TRAF2, E2F1) with more than 7 connections. Also, the same nodes showed the higher betweenness score. The network based on the differentially expressed genes related to stress identified one highly connected node (H2AFX) with more than 7 connections and the higher betweenness score. No relevant networks were formed between differentially expressed genes related to mitochondrion.

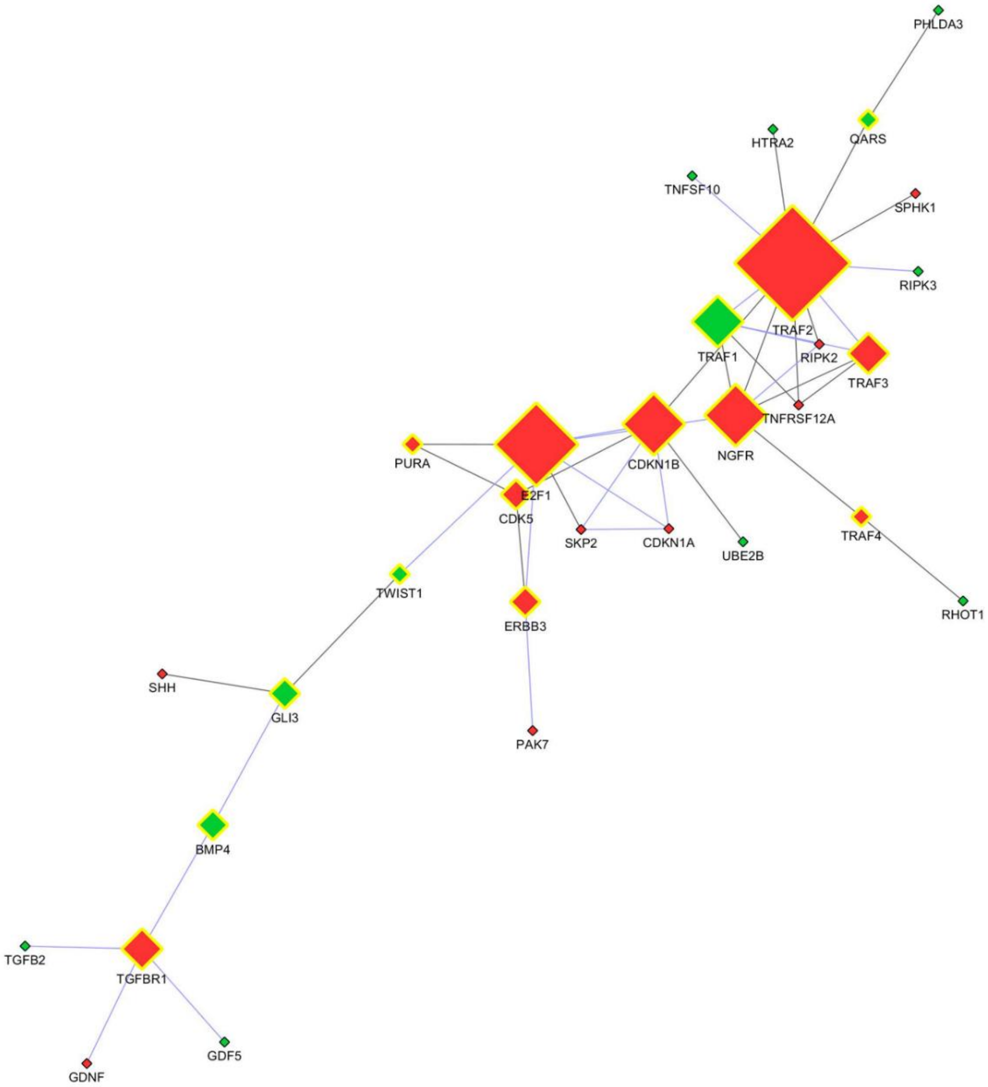

Figure S2. Protein interaction network showing interactions between differentially expressed genes related to death in sciatic nerve from 60-day-old presymptomatic SOD1^{G93A} mice. Up and down-regulated genes are represented respectively as red and green diamonds. Nodes with the highest values for node degree (number of connections) and node betweenness (number of shortest paths) are represented with a yellow border. Pathway and physical interactions are depicted respectively as grey and blue edges. The genes *E2f1*, *Ngfr*, *Gli3*, *Erbb3* and *Cdk5* or their related products were already described in the context of Amyotrophic Lateral Sclerosis.

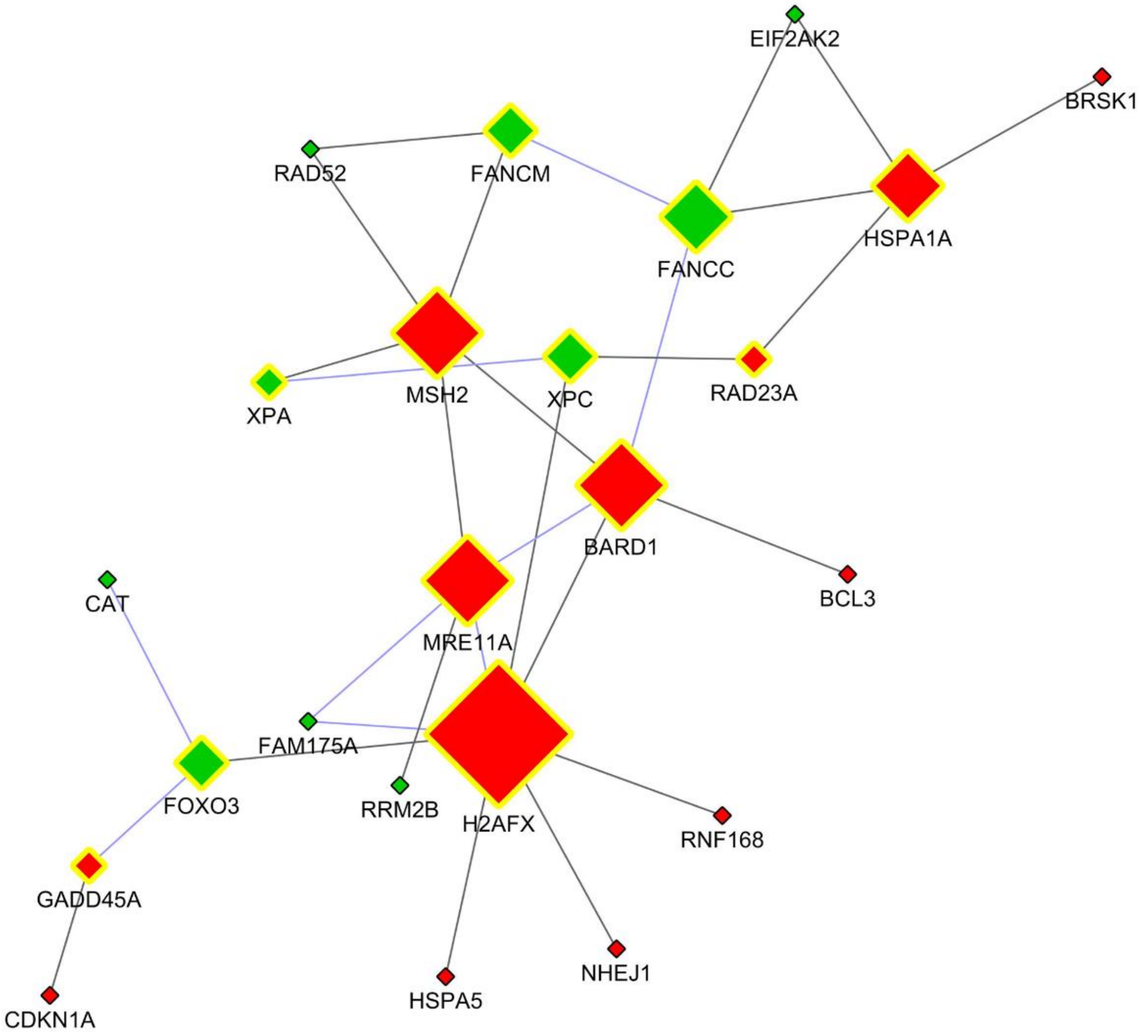

Figure S3. Protein interaction network showing interactions between differentially expressed genes related to stress in sciatic nerve from 60-day-old presymptomatic SOD1^{G93A} mice. Up and down-regulated genes are represented respectively as red and green diamonds. Nodes with the highest values for node degree (number of connections) and node betweenness (number of shortest paths) are represented with a yellow border. Pathway and physical interactions are depicted respectively as grey and blue edges. The gene *Foxo3* or their related products were already described in the context of Amyotrophic Lateral Sclerosis.

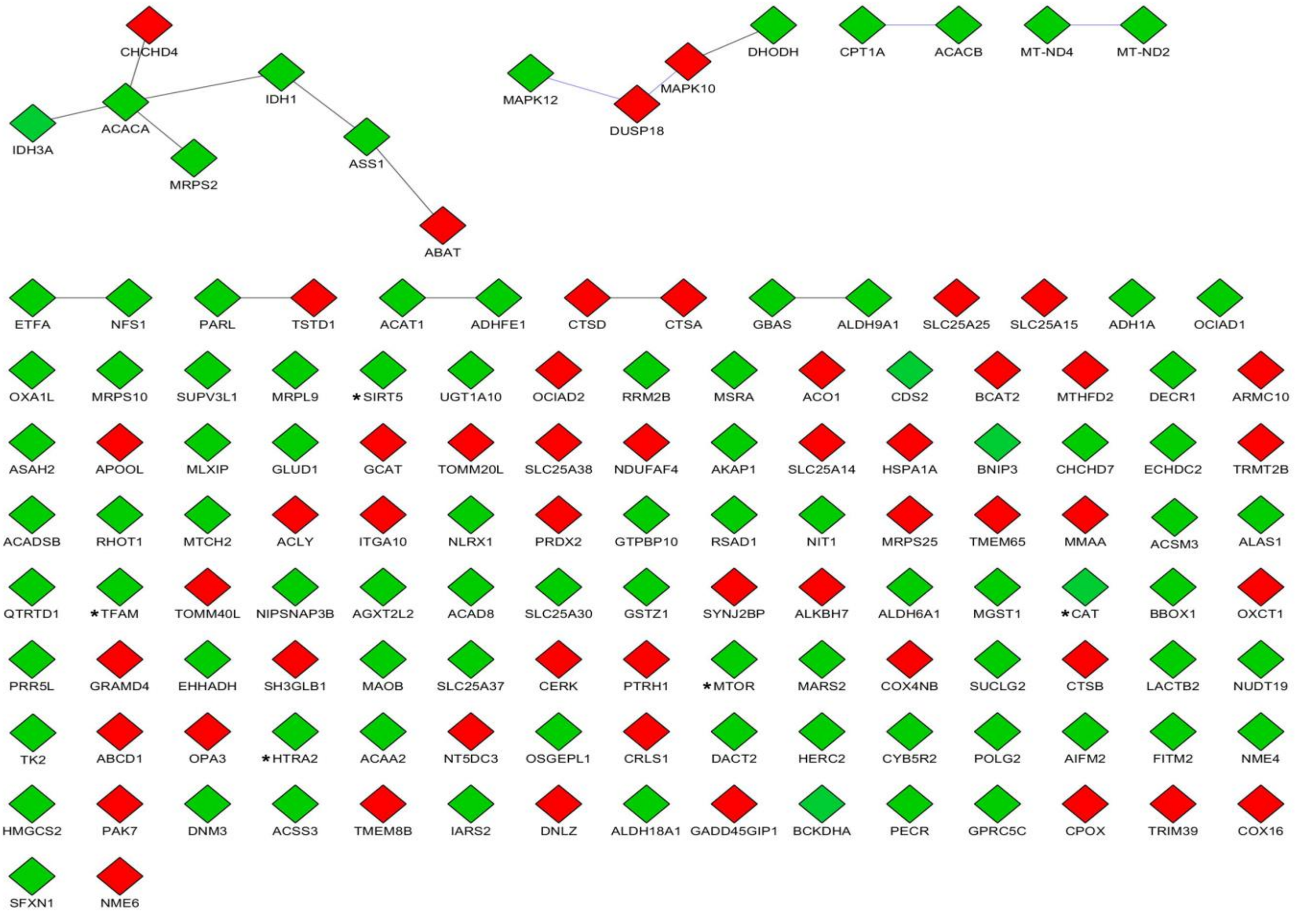

Figure S4. Protein interaction network showing interactions between differentially expressed genes related to mitochondrion in sciatic nerve from 60-day-old presymptomatic SOD1^{G93A} mice. Up and down-regulated genes are represented respectively as red and green diamonds. Pathway and physical interactions are depicted respectively as grey and blue edges. The genes pointed by * (*Sirt5, Tfam, Htra2, Mtor, Cat)* or their related products were already described in the context of Amyotrophic Lateral Sclerosis.

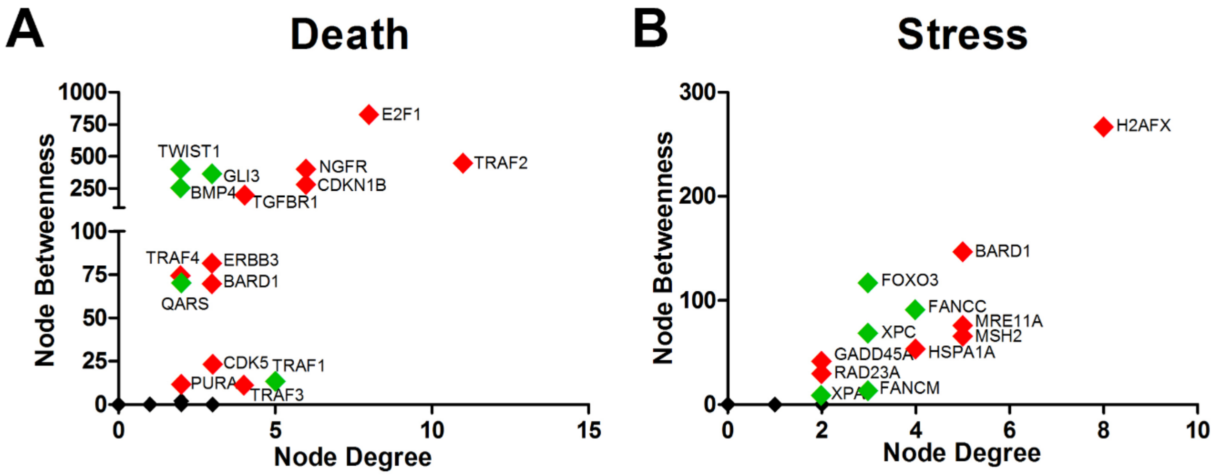

Figure S5. Scatter plot showing the correlation between highest values of node degree (hubs) and node betweenness (bottlenecks) for each differentially expressed genes related to death (A) and stress (B) in sciatic nerve from 60-day-old presymptomatic SOD1^{G93A} mice. Up and down-regulated genes are represented respectively as red and green diamonds.

Supplementary Material References

Bajaj, N.P. (2000). Cyclin-dependent kinase-5 (CDK5) and amyotrophic lateral sclerosis. *Amyotroph Lateral Scler Other Motor Neuron Disord* 1, 319-327

Crippa, V., Boncoraglio, A., Galbiati, M., Aggarwal, T., Rusmini, P., Giorgetti, E., Cristofani, R., Carra, S., Pennuto, M., and Poletti, A. (2013a). Differential autophagy power in the spinal cord and muscle of transgenic ALS mice. *Front Cell Neurosci* 7, 234. doi: 10.3389/fncel.2013.00234

Crippa, V., Galbiati, M., Boncoraglio, A., Rusmini, P., Onesto, E., Giorgetti, E., Cristofani, R., Zito, A., and Poletti, A. (2013b). Motoneuronal and muscle-selective removal of ALS-related misfolded proteins. *Biochem Soc Trans* 41, 1598-1604. doi: 10.1042/BST20130118

Crippa, V., Sau, D., Rusmini, P., Boncoraglio, A., Onesto, E., Bolzoni, E., Galbiati, M., Fontana, E., Marino, M., Carra, S., Bendotti, C., De Biasi, S., and Poletti, A. (2010). The small heat shock protein B8 (HspB8) promotes autophagic removal of misfolded proteins involved in amyotrophic lateral sclerosis (ALS). *Hum Mol Genet* 19, 3440-3456. doi: 10.1093/hmg/ddq257

Dobrowolny, G., Aucello, M., Molinaro, M., and Musaro, A. (2008). Local expression of mIgf-1 modulates ubiquitin, caspase and CDK5 expression in skeletal muscle of an ALS mouse model. *Neurol Res* 30, 131-136. doi: 10.1179/174313208X281235

Kriz, J., Gowing, G., and Julien, J.P. (2003). Efficient three-drug cocktail for disease induced by mutant superoxide dismutase. *Ann Neurol* 53, 429-436. doi: 10.1002/ana.10500

Nguyen, M.D., Lariviere, R.C., and Julien, J.P. (2001). Deregulation of Cdk5 in a mouse model of ALS: toxicity alleviated by perikaryal neurofilament inclusions. *Neuron* 30, 135-147. doi: S0896-6273(01)00268-9

Park, K.H., and Vincent, I. (2008). Presymptomatic biochemical changes in hindlimb muscle of G93A human Cu/Zn superoxide dismutase 1 transgenic mouse model of amyotrophic lateral sclerosis. *Biochim Biophys Acta* 1782, 462-468. doi: 10.1016/j.bbadis.2008.04.001
